# Chronic choline restriction remodels hepatic lipid metabolism and drives insulin resistance through a CD36-ETNPPL regulatory axis

**DOI:** 10.1016/j.molmet.2026.102411

**Published:** 2026-07-14

**Authors:** Evan M. Paules, Blake Rushing, Israel Aguilar-Ordoñez, Jose L. Garduno-Hernandez, Ariana Reid, Barbara J. Davis, Teodoro Bottiglieri, Karel Kalecký, Erik Lopez-Gallardo, Stephen D. Hursting, Isis Trujillo-Gonzalez

**Affiliations:** 1 Nutrition Research Institute, University of North Carolina at Chapel Hill, Kannapolis, NC, USA; 2 Department of Nutrition, Gillings School of Global Public Health, University of North Carolina at Chapel Hill, Chapel Hill, NC, USA; 3 Tecnologico de Monterrey, oriGen Project, Monterrey, Nuevo Leon, Mexico; 4 Center of Metabolomics, Institute of Metabolic Disease, Baylor Scott and White Research Institute, Dallas, TX 75204, USA; 5 Advanced Center for Chronic Disease (ACCDiS), Department of Biochemistry and Molecular Biology, Faculty of Chemical and Pharmaceutical Sciences & Faculty of Medicine, University of Chile, Santiago, Chile; 6 Lineberger Comprehensive Cancer Center, University of North Carolina at Chapel Hill, Chapel Hill, NC, USA

**Keywords:** Choline, Metabolic syndrome, *Cd36*, ETNPPL, Insulin resistance

## Abstract

Chronic choline insufficiency reprograms hepatic metabolism and drives insulin resistance independent of obesity. While complete choline deficiency causes liver injury, the metabolic consequences of sustained, suboptimal intake, observed in ∼90% of US adults, remain poorly defined. Here, we used integrated lipidomic, metabolomic, and transcriptomic profiling to determine how graded choline intake (0.5, 1.4, or 6.3 g/kg) regulates hepatic metabolism during a control (Con) or high-fat (HF) diet-induced obesity regimen. Under Con diets, low choline intake induced a distinct metabolic state characterized by remodeled hepatic lipid architecture, particularly within triglyceride and glycerolipid species, without altering bulk triglyceride accumulation. Mechanistically, low choline disrupted phospholipid balance and induced a coordinated, sex-dependent transcriptional response, identifying ethanolamine-phosphate phospho-lyase (ETNPPL) and the fatty acid transporter CD36 as top choline-responsive genes. These metabolic effects were unique to the Con low choline group, as a high-fat diet masked all choline-dependent variations. Specifically, ETNPPL protein abundance increased under low choline Con conditions in males but not females. Functionally, this sustained restriction led to progressive hyperglycemia and insulin resistance exclusively in male mice, whereas females remained metabolically protected. Together, these findings demonstrate that chronic choline restriction remodels hepatic lipid metabolism in the absence of obesity and define a CD36–ETNPPL axis linking choline availability to sex-specific insulin resistance.

## Highlights

• Chronic low choline intake drives insulin resistance in male mice but not female mice independent of obesity.
• Multi-omics integration identifies ETNPPL as a key choline-responsive regulator of hepatic lipid remodeling.
• Choline restriction remodels hepatic lipid composition without increasing total triglyceride content.
• High-fat diet obscures graded, choline-dependent metabolic effects in the liver.

## Graphical abstract

**Figure:**
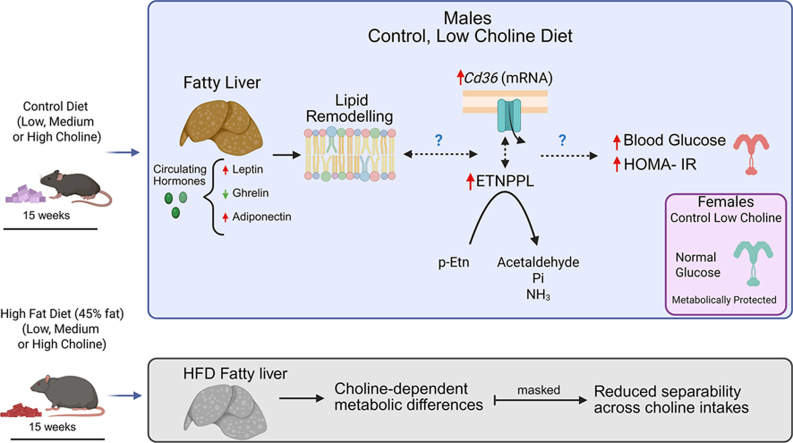

## 1 Introduction

Insulin resistance (IR) is a central driver of cardiometabolic disease. Although frequently associated with obesity, IR also occurs in nonobese individuals, indicating that nutrient composition independent of excess adiposity can influence cardiometabolic disease risk and progression [1]. Micronutrient availability shapes lipid handling, endocrine signaling, and transcriptional regulation in ways that remain incompletely defined. Choline is an essential nutrient that integrates phosphatidylcholine (PtdCho) synthesis with one-carbon metabolism, placing it at a critical intersection between membrane phospholipid homeostasis and methyl donor availability [2]. In experimental rodent models, complete dietary choline deprivation or genetic disruption of PtdCho synthesis pathways leads to severe hepatic injury and steatosis, largely due to impaired very-low-density lipoprotein (VLDL) export secondary to insufficient PtdCho synthesis [[3], [4], [5]–5].

However, complete elimination of dietary choline does not reflect typical human intake patterns, in which chronic suboptimal intake is common whereas absolute deprivation is exceedingly rare [6]. Clinical studies using depletion-repletion designs have generally provided <50 mg/day, a level at which 77% of men and 80% of postmenopausal women developed fatty liver or muscle damage that resolved following graded repletion at 25%, 50%, 75%, and 100% of the adequate intake (AI) [7].

At the upper end of restriction, moderate intake of approximately 300 mg/day in healthy men did not produce elevations in liver enzymes over 12 weeks [8], while observational data from free-living populations suggest that intakes below ∼7 mg/kg body weight are associated with increased odds of hepatic steatosis [9]. Together, these data suggest a range across which hepatic responses to low choline intake transition from undetectable to overt dysfunction and establish that these responses are dose-dependent and reversible. However, they do not capture the metabolic consequences of moderate, sustained reductions in choline intake that more closely resemble habitual human diets.

The metabolic consequences of chronic low choline intake under nonobese conditions remain poorly characterized. Preclinical work has focused on hepatic lipid accumulation and cholesterol homeostasis using choline supplementation, methionine-choline deficienct (MCD) models [[10], [11], [12]–12], or choline-only restriction with graded methionine [13]. Across these models, the severity of hepatic injury depends on both the duration and the presence of accompanying metabolic stressors, such as methionine deficiency or high-fat intake. Steatosis develops within 2–4 weeks under MCD conditions but requires 12 weeks under a high-fat, choline-only restriction with adequate methionine [13]. However, these models do not address the consequences of moderate, sustained choline reduction in the absence of methionine deficiency or obesity, the scenario that most closely resembles habitual human intake.

Hepatic fatty acid flux and phospholipid remodeling represent central mechanisms through which nutrient insufficiency disrupts insulin sensitivity [[14], [15], [16]–16]. Dysregulation of lipid uptake and oxidation promotes the accumulation of oxidized triglycerides and bioactive lipid intermediates that impair hepatic insulin signaling and promote systemic insulin resistance [[17], [18], [19]–19]. Concurrent alterations in phospholipid metabolism can remodel membrane composition and reprogram transcriptional networks governing metabolic homeostasis. Together, these coordinated lipid perturbations provide a mechanistic framework by which sustained low choline intake may drive systemic insulin resistance and cardiometabolic disease progression.

Herein we examined the metabolic consequences of sustained low dietary choline intake under non-obesogenic conditions and in a high-fat diet context (HF). Under HF, the expected metabolic phenotype predominated and masked graded choline-dependent effects. In the context of the control diet, we show that low choline drives systemic insulin resistance and hepatic injury in male (but not female) mice, characterized by steatosis, inflammatory features, and disrupted endocrine signaling. Multi-omic profiling revealed extensive remodeling of hepatic lipid composition, including accumulation of oxidized lipid species, alongside coordinated transcriptional reprogramming. Integrative analysis identified a CD36-ethanolamine phosphate phospho-lyase (ETNPPL) axis as a choline-sensitive regulator linking phospholipid remodeling to hepatic metabolic dysfunction.

## 2 Methods

### 2.1 Animals, diets, and experimental design

All protocols were reviewed and approved by the Institutional Animal Care and Use Committee (IACUC protocol number: 23-005) at the North Carolina Research Campus in Kannapolis, NC. C57BL/6J mice (98 males, 63 females; 8 weeks of age) were purchased from the Jackson Laboratory (JAX Strain #000664) and housed at 24 °C with a 12-hour light/dark cycle (lights on from 7:00 to 19:00, with 7:00 defining Zeitgeber time 0), given unrestricted access to water, and fed a control, medium choline (ConMC) diet ad libitum for 2 weeks. Mice obtained from the Jackson Laboratory were previously maintained on LabDiet® 5K52 (1.2 g/kg choline) for production and breeding; upon arrival, all mice were transitioned to the medium choline diet (1.4 g/kg choline chloride) for two weeks of acclimation prior to randomization. Because preliminary analyses indicated a stronger metabolic response to graded choline intake in males, an additional male cohort was included to increase statistical power for mechanistic analyses and to obtain sufficient tissue for multi-omic profiling. Female mice were analyzed in parallel, and these data are presented as secondary analyses.

Following 2 weeks of acclimation, the 11-week-old mice were block randomized, based on baseline body weight, to one of 6 different diet groups: ConLC (10 kcal% fat; Research Diets #D22061507; 0.5 g/kg choline), ConMC (10 kcal% fat; Research Diets #D22061508; 1.4 g/kg choline), ConHC (10 kcal% fat; Research Diets #D22061509; 6.3 g/kg choline), HFLC (45 kcal% fat; Research Diets #D22061504; 0.5 g/kg choline), HFMC (45 kcal% fat; Research Diets #D22061505; 1.4 g/kg choline), and HFHC (45 kcal% fat; Research Diets #D22061506; 6.3 g/kg choline), all fed ad libitum for 12 weeks. All choline concentrations are expressed as choline chloride. Certificates of Conformance are provided as Supplementary File 1. The three diets were designed to span the physiologically relevant range of choline intake. The medium choline diet (1.4 g/kg choline chloride, ∼1.04 g/kg choline) served as the adequate reference. This choline content matches the AIN-93 standard rodent diet (2.5 g/kg choline bitartrate, 1.03 g/kg choline) and approximates the choline AI for adults (550 mg/day). The low choline diet (0.5 g/kg choline chloride) provides approximately 0.36 times the adequate level, modeling sustained sub-adequate choline intake within the lower range of consumption. The high choline diet (6.3 g/kg choline chloride) provides 4.5 times the adequate diet, the supplementation ratio used in prior choline studies [[20], [21], [22]–22]. This diet model choline supplementation in the supraphysiologic range, with the Tolerable Upper Intake Level (3.5 g/day) established for adults by the U.S. Institute of Medicine. Additionally, all diets maintained constant levels of methyl-donor nutrients other than choline. The experimental diets were iterations of Research Diets' high-fat formulation for diet-induced obesity studies (D12451) and its corresponding control diet (D12450H), with l-cystine (3 g/kg, 12 kcal) and casein, the protein source (200 g/kg, 800 kcal), held constant across all groups. No methionine was added beyond that naturally present in casein (2.9 g per 100 g casein, equating to ∼5.8 g/kg in each experimental diet), which was therefore also constant across groups. This design held the dietary supply of methionine and cysteine uniform across all diets, ensuring that choline was the only methyl-donor source that was deliberately varied between the low, medium, and high choline diets. Full diet details can be found in Supplementary Table 1.

Terminal blood collection was performed using a cardiac puncture under deep anesthesia (3–4% isoflurane inhalation with confirmation of deep anesthesia via toe pinch) was followed by euthanasia via cervical dislocation. All tissues were weighed using a calibrated scale and either fixed (10% W/V neutral buffered formaldehyde) or flash frozen in liquid nitrogen for downstream analysis. Humane endpoints included weight loss greater than 20% within a week, signs of significant distress (ruffled coat or hunched posture), or disease. Sample size was determined by using a power analysis using G∗Power ver. 3.1.9.7 [23], based on pilot data using body weight as the primary outcome measure. The analysis assumed a Cohen's effect size 0.36, with *α* = 0.05, and power = 0.80. Group sizes were as follows: ConLC (males n = 15, females n = 13), ConMC (males n = 17, females n = 9), ConHC (males n = 17, females n = 11), HFLC (males n = 17, females n = 10), HFMC (males n = 17, females n = 10), and HFHC (males n = 15, females n = 10).

### 2.2 Metabolic phenotyping

Mice were fasted 4 h prior to all procedures. This duration was selected to clear postprandial nutrient absorption from the gastrointestinal tract while preserving hepatic glycogen stores minimizing the metabolic stress of prolonged fasting. Longer or overnight fasts progressively deplete hepatic glycogen and promote weight loss and catabolic responses that increase inter-animal variability in glucose and metabolic measurements [[24], [25], [26]]. The 4-hour fast was applied uniformly across all metabolic assessments to ensure internal consistency and comparability among groups. All experiments were performed at a consistent time of day through the study. Body weight and food intake were recorded three times per week (Monday, Wednesday, and Friday) using a calibrated scale. Body composition, including fat mass and lean mass, was measured at baseline and immediately prior to euthanasia using an EchoMRI-100H analyzer (EchoMRI LLC, Houston, TX, USA).

At euthanasia, blood was collected by terminal cardiac puncture into K2EDTA-coated microtainer® tubes (BD, Franklin Lakes, NJ) for plasma isolation. Tubes were kept on ice and centrifuged at 2,000 × rcf for 10 min at 4 °C, and plasma was stored at −80 °C until analysis. Plasma concentrations of ghrelin, glucose-dependent insulinotropic polypeptide (GIP), glucagon-like peptide-1 (GLP-1), insulin, leptin, plasminogen activator inhibitor-1 (PAI-1), resistin, glucagon, and adiponectin were quantified using a Bio-Plex Multiplex Immunoassay (Bio-Plex Pro Mouse Diabetes 8-Plex Assay; Bio-Plex Pro Mouse Diabetes Adiponectin Assay) and analyzed with a Bio-Plex MAGPIX Multiplex Reader (Bio-Rad; Hercules, CA, USA). HOMA-IR was calculated from fasting glucose and insulin concentrations as previously described [27]. Blood glucose was measured using a tail nick procedure at baseline, week 5, week 10, and week 15 (prior to euthanasia), using a OneTouch Ultra 2 glucometer (LifeScan, Malvern, PA, USA).

### 2.3 Histology and NAFLD scoring

Hepatic tissue from the left lobe was fixed in 10% W/V neutral buffered formaldehyde for 48 h and then transferred to 70% ethanol until processing. Sectioning was performed by the Histology Research Core Facility at UNC-Chapel Hill. Sections were paraffin embedded and cut using a microtome (6 μm thickness) in the transverse plane. Three sections per liver were stained with either hematoxylin and eosin (H&E) or Sirius Red. Labeled slides 2–72 consisting of 3 consecutive sections per liver were evaluated and scored for steatosis according to criteria established by Kleiner et al. [28]. Scoring was performed blinded to treatment group by a pathologist. All sections were independently reviewed and graded on at least three separate occasions by an experienced veterinary pathologist to ensure consistency and reproducibility. Steatosis graded 0 was <5% of liver involvement even if some evidence was observed, and grades 1–3 corresponded to 5–33%, 33–66% and >66% of parenchymal involvement, respectively. Fibrosis was assessed on Sirius Red stained sections. The NAFLD Activity Score (NAS) was calculated by summing the scores for hepatic steatosis, inflammation, and ballooning. All slides were also fully documented in support of the pathological report, with 2–3 images captured per slide (3 slides H&E, 3 slides Sirius Red per liver) yielding >1,200 images. The complete full-resolution image set has been deposited in the UNC Dataverse, with access provided on request through the corresponding author.

### 2.4 Hepatic lipid quantification

#### 2.4.1 Triglyceride assay

Triglyceride levels were determined using the Cayman Chemical Triglyceride Colorimetric Assay Kit (Item No. 10010303) according to the manufacturer's instructions. Frozen liver tissue (10–20 mg), was extracted in chloroform:isopropanol:igepal (7:11:0.1), and homogenized using a TissueLyser LT (Qiagen, Cat# 85600) with steel beads. Samples were centrifuged, and the solvent evaporated using a speedvac. Lipid extracts were solubilized in NP40 Substitute Assay Reagent (1X) and heated briefly at 100 °C to maximize solubilization. Standards, samples, and controls were assayed in duplicate in a 96-well plate format. Ten microliters of each standard or sample were added to designated wells, followed by 150 μL of the Triglyceride Enzyme Mixture. Plates were incubated for 60 min at room temperature, and absorbance was measured at 540 nm. Triglyceride concentrations were calculated from a standard curve generated by linear regression of corrected absorbance values.

### 2.5 Targeted metabolomics

Targeted metabolomics was performed using the MxP Quant 500 XL kit (Biocrates Life Sciences AG, Innsbruck, Austria). Pulverized liver tissue was extracted in isopropanol (3 μl/mg) and ultrasonically homogenized. Homogenates were centrifuged at 15,000 × *g* for 10 min at 4 °C, and 10 μL of the supernatant was transferred to each of two kit 96-well filter plates preloaded with internal standards. Samples were dried under nitrogen, derivatized with phenylisothiocyanate (PITC), dried again, and extracted with 300 μL of 5 mM ammonium acetate in methanol. Extracts were collected into a deep-well plate for LC-MS/MS and FIA-MS/MS analysis. LC-MS/MS was used for chromatographic separation and quantification of fatty acids and non-lipid small molecules (106 variables), while FIA-MS/MS was employed for complex lipids and the sum of hexose sugars (913 variables). Analyses were performed on a triple quadrupole mass spectrometer QTRAP 7500 (AB Sciex LLC, Marlborough, MA, USA) equipped with an electrospray ionization source in multiple reaction monitoring (MRM) mode. Chromatographic peaks were identified, reviewed, and integrated in the in-house software Integrator (patent pending; Application # PCT/US24/51426).

Data analysis of metabolomic data was done in R v4.4.2 with RStudio v2024.12.0. Areas of metabolite peaks were divided by areas of their respective internal standards. For most compounds, concentrations were estimated linearly from expected concentrations in the kit quality control samples using their median. Seven-point calibration was applied where possible (standards included for 42 comopunds). Limits of detection (LODs) were calculated as 3 × median values in blanks. Metabolites with more than 50% values below LOD in all choline intake level groups and main diet groups were filtered out. Metabolic indicators were calculated according to Biocrates MetaboINDICATOR formulas. Ratios with zeros were treated as missing values and not included in the analysis. Additional data transformations were applied: To better approximate Gaussian distributions, we applied Box–Cox transformation with R package *car*. Tukey's fencing was used to adjust remote outliers (k = 3) to protect against skewing the means by extreme values while not reducing the variance greatly compared to outlier removal. PCA analysis was performed with R package *ropls* and group clusters were plotted with R package *ggpubr* and compared with two-sample Hotelling's t-squared test from R package *Hotelling* over the principal components. Heatmaps with Ward's hierarchical clustering were plotted with R package *gplots* after selecting top features according to ANOVA p-values (minimum of 20 features with smallest p-values regardless of significance, up to 50 features when p-value ≤0.05).

### 2.6 mRNA-sequencing and differential expression analysis

Total RNA was isolated from the liver using a Purelink RNA mini kit (Invitrogen, Waltham, MA, USA; Cat#12183018A) following the manufacturer's instructions. Briefly, liver tissue was homogenized in lysis buffer using a TissueLyser LT (Qiagen, Venlo, Limburg, Netherlands; Cat# 85600) with stainless steel beads. Resulting homogenates were centrifuged and applied to silica spin columns for RNA binding. RNA concentrations and integrity were assessed using a 4150 TapeStation System (Agilent, Santa Clara, California, USA; Cat# G2992AA). RNA-seq libraries were prepared by Azenta Life Sciences using a standardized workflow appropriate for sample type and study goals. Briefly, poly(A) selection was performed to enrich for mRNA, followed by RNA fragmentation, first- and second-strand cDNA synthesis, end repair/A-tailing, adapter ligation, and PCR amplification with unique indices. Library quality and size distribution were assessed by microfluidic electrophoresis, and libraries were quantified by qPCR and/or fluorometry prior to pooling. Pooled libraries were sequenced by Azenta on an Illumina NovaSeq 6000 to generate paired-end reads at a target depth of 30 million reads. Base calling and demultiplexing were performed using Illumina software, and FASTQ files were returned for downstream analysis.

The bioinformatics code used to perform differential expression and downstream analyses is publicly available at https://github.com/Iaguilaror/itrujillo-ctp-dio-choline/tree/main. Conversion of FASTQ files to gene-level count tables was performed using the nf-core/rnaseq pipeline (v3.18.0-gb96a753) with default parameters, using the GRCm38 mouse reference genome from iGenomes (https://support.illumina.com/sequencing/sequencing_software/igenome.html). Briefly, adapter and quality trimming were conducted with Trim Galore, and reads shorter than 20 bp after trimming were discarded. Read quality was assessed using FastQC, and summary reports were generated with MultiQC. Transcript quantification was performed with Salmon against the mouse reference transcriptome and corresponding Ensembl gene annotation (GTF). Only gene-level Salmon counts were retained for downstream analyses, including normalization and differential expression testing. Differential expression analysis was performed using the nf-core/differentialabundance pipeline (v1.5.0-g3dd360f) with default parameters. The salmon.merged.gene_counts.tsv file served as input for each contrast reported in the manuscript. The pipeline validated input consistency, normalized count data, and conducted differential expression testing using DESeq2, which models raw counts using negative binomial generalized linear models. Outputs included tabulated differential expression results and an HTML report summarizing data quality, normalization, and model performance. Differentially expressed genes (DEGs) were defined using an adjusted p-value <0.1 and an absolute log_2_ fold change >0.5.

### 2.7 Western blot analysis

Total liver protein lysates were prepared using RIPA buffer supplemented with protease and phosphatase inhibitor cocktail (Thermo Fisher, Waltham, MA, USA; Cat# 78444). Liver tissue was homogenized using a TissueLyser LT (Qiagen, Cat# 85600) with stainless steel beads. Homogenates were centrifuged and protein concentrations were determined using a Bradford assay (Bio-Rad, Cat# 5000006) according to the manufacturer's instructions. Samples were mixed with Laemmli sample buffer and boiled for 5 min. For SDS-PAGE, proteins were separated on 10% polyacrylamide gels under reducing conditions, and equal amounts of protein were loaded per lane. Proteins were transferred to nitrocellulose membranes using a standard wet-transfer protocol. Membranes were blocked for 1 h at room temperature using StartingBlock™ T20 Blocking Buffer (Thermo Fisher, Cat# 37538) and incubated overnight at 4 °C with primary antibodies diluted in blocking buffer, including CD36 (Abcam, ab124515), Etnppl (Thermo Fisher, PA5-104423), and *β*-actin (Santa Cruz Biotechnology, sc-130065) as a loading control. After washing, membranes were incubated for 1 h at room temperature in the dark with fluorescent secondary antibodies (LI-COR Biosciences, Lincoln, NE; 926–68070 and 926–32211). Blots were imaged using a LI-COR Odyssey imaging system, and band intensities were quantified using Image Studio software (LI-COR). Target protein levels were normalized to *β*-actin.

### 2.8 Multi-omic integration and pathway analysis

Relative abundance data from hepatic metabolomics and transcriptomics datasets were imported into OmicsAnalyst for integrated multivariate analysis, with dietary choline intake included as metadata [29]. All datasets were autoscaled to harmonize distribution patterns prior to analysis. Principal component analysis (PCA) was first performed on each individual omics layer, followed by integrated analysis using Data Integration Analysis for Biomarker discovery using Latent variable approaches for Omics studies (DIABLO), a supervised multivariate method based on multi-block partial least squares–discriminant analysis (PLS-DA) that identifies features contributing to class separation in high-dimensional datasets [30]. DIABLO was used to identify variables differentiating the low, medium, and high dietary choline intake on loading scores. For visualization and downstream integration, non-lipid features were summarized into composite indicators to reduce dimensionality and capture pathway level variation, whereas lipidomic data were retained at the individual species level to preserve biologically meaningful differences in lipid composition. Metabolites, transcripts, and proteins identified by DIABLO were subsequently subjected to joint pathway analysis using MetaboAnalyst 5.0 for integrated metabolite-transcript pathway analysis [31]. xOmicsShiny was used to generate volcano plots comparing low and high choline intakes for each omics layer [32]. Variable importance in projection (VIP) scores derived from multivariate analyses performed in MetaboAnalyst were used to rank metabolites, lipids, or transcripts based on their contribution to group separation. Variables with a VIP >1.0 were selected as top discriminatory features. Pathway enrichment for metabolites and transcripts was performed in MetaboAnalyst, whereas lipid pathway enrichment analysis was performed using LipidLION [33].

Ingenuity Pathway Analysis (IPA; QIAGEN) was used to perform multi-omic pathway enrichment, upstream regulator analysis, and network modeling. Differentially expressed genes and/or metabolites identified from upstream analyses were uploaded into IPA along with corresponding fold-change values and statistical significance metrics. Analyses were conducted using the Ingenuity Knowledge Base as the reference set, restricted to experimentally observed relationships. Core Analysis was performed to identify significantly enriched canonical pathways, upstream regulators, and molecular interaction networks. Pathway significance was assessed using right-tailed Fisher's exact tests, while activation z-scores were used to predict the directionality of pathway and regulator activity. Only pathways and regulators meeting significance thresholds (p < 0.05) were considered. Networks were generated based on direct and indirect molecular interactions, with network scores reflecting the likelihood of the observed gene set occurring by chance.

### 2.9 Statistical analysis

Outliers were determined by a ROUT test. Cleaned data was assessed for normality using a Shapiro–Wilks test followed by either a one-way ANOVA, Welch, two-way ANOVA, mixed effects model, or Kruskal–Wallis test depending on the data structure. Based on the normality of the data, either a Tukey, Dunnett's, Dunn's, or Fisher's LSD post hoc test was conducted where applicable. Data are displayed as means ± standard error in all figures and significance was called at p < 0.05. Statistical tests used for each analysis can be found in the respective figure legend. Complete statistical parameters for all reported comparisons, including omnibus test statistics with degrees of freedom and post hoc test statistics, are provided in Supplementary Table 2.

## 3 Results

### 3.1 Sustained low choline intake selectively increases body weight in male but not female mice

**Figure 1:**
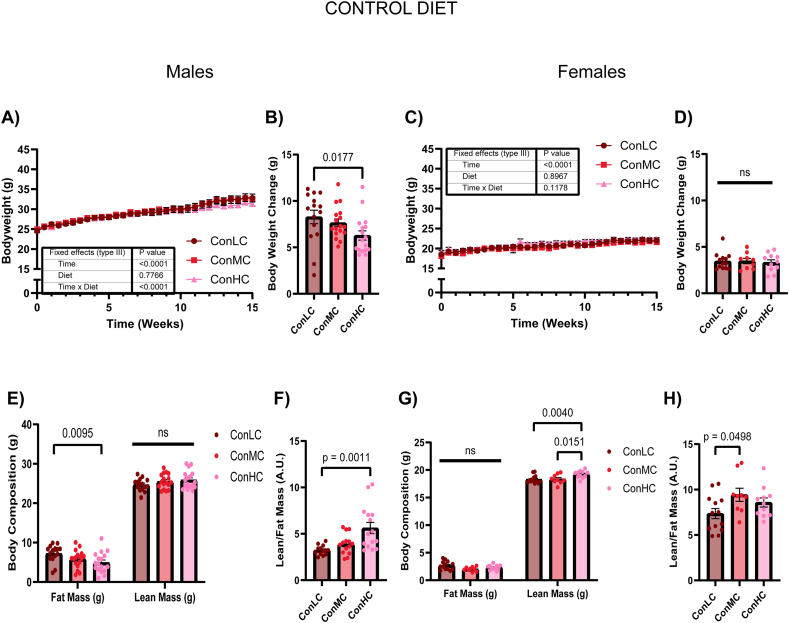
**Low choline intake increases adiposity and reduces lean mass in male (but not female) mice under nonobese conditions.** Body weight over time with corresponding mixed-effects models and body weight change from baseline to study termination in males (**A, B**) and females (**C, D**) with low (LC), medium (MC), and high (HC) choline intakes. Body composition and Lean/Fat Mass ratio for males (**E, F**) and female (**G, H**). Sample sizes per group: ConLC (males *n* = 15, females *n* = 13), ConMC (males *n* = 17, females *n* = 9), and ConHC (males *n* = 17, females *n* = 11). A Mixed-effects analysis followed by a Tukey multiple comparisons test was performed for (**A**) and (**C**). A two-way ANOVA followed by a Tukey multiple comparisons test was performed for (**E**) and (**G**). A Kruskal–Wallis one-way ANOVA was performed followed by a Dunn's multiple comparisons test for (**B**), (**F**), and (**H**). A lognormal one-way ANOVA was performed followed by a Tukey's multiple comparisons for (**D**). Significance was set at *p* < 0.05. Data are presented as mean ± SEM, with individual data points representing single animals.

To determine the metabolic consequences of graded dietary choline intake under different choline intakes, 82C57BL/6J mice (49 males, 33 females) were randomized to receive either a Con diet low (ConLC; males n = 15, females n = 13), medium (ConMC; males n = 17, females n = 9), or high (ConHC; males n = 17, females n = 11) choline for 15 weeks. (Suppl. Fig. 1A). When analyzed across sexes, neither baseline body weight nor longitudinal body weight trajectories differed among dietary groups **(**Suppl. Fig. 1B and C). Given well-established sexual dimorphism in choline metabolism [34] and cardiometabolic regulation [35], metabolic outcomes were analyzed separately in males and females. Males exhibited a significant time × diet interaction by mixed-effects modeling **(p < 0.0001**), indicating differential body weight trajectories across choline levels (Figure 1A). Analysis of body weight change from baseline to study end revealed greater weight gain in ConLC compared to ConHC males (**p = 0.0177**) (Figure 1B).

In contrast females demonstrated significant weight gain over time (**p < 0.0001**) but no diet effect or time × diet interaction was observed (Figure 1C). Body weight change did not differ among choline groups in females (Figure 1D). There were no differences in food intake across choline groups (Suppl. Fig. 1D and E), including between low, medium and high choline groups within males and within females, indicating that body weight divergence was not driven by altered caloric intake. These data identify a male-specific weight divergence in response to sustained low choline that is independent of food intake. To define the underlying phenotype, we next assessed body composition.

### 3.2 Low choline intake alters lean-fat balance

Using EchoMRI, we quantified fat and lean mass to determine the compositional basis of the observed weight divergence. In males, ConLC mice had significantly more fat mass than ConHC mice (**p = 0.0095**), with no significant differences in lean mass (Figure 1E). Male ConLC mice also had a lower lean-to-fat mass ratio, a sensitive indicator of metabolic health, compared to ConHC (**p = 0.0011**) (Figure 1F). These data indicate that sustained low choline intake may be shifting body composition toward a less favorable metabolic profile in males despite modest differences in total mass.

In females, fat mass did not differ across choline levels, but lean mass tracked choline availability, ConHC females had significantly greater lean mass than both ConLC (**p = 0.004**) and ConMC (**p** = **0.0151**) females (Figure 1G). Consistent with this, ConLC females had a lower lean-to fat mass ratio compared to ConMC females (**p = 0.0498**) (Figure 1H). The female response to low choline therefore appeared in the lean compartment rather than the fat compartment. The opposite of what we observed in males.

These data indicate that low choline intake reshapes body composition primarily in males, where it increases fat mass and lowers the lean-to-fat mass ratio, consistent with the male weight divergence described above. In females, these effects were more limited, lean mass was modestly lower in the low choline animals, with a corresponding reduction in the lean-to-fat mass ratio, and no change in fat mass or body weight.

### 3.3 Choline intake has limited, sex-specific effects on tissue weights

**Supplementary Figure 2:**
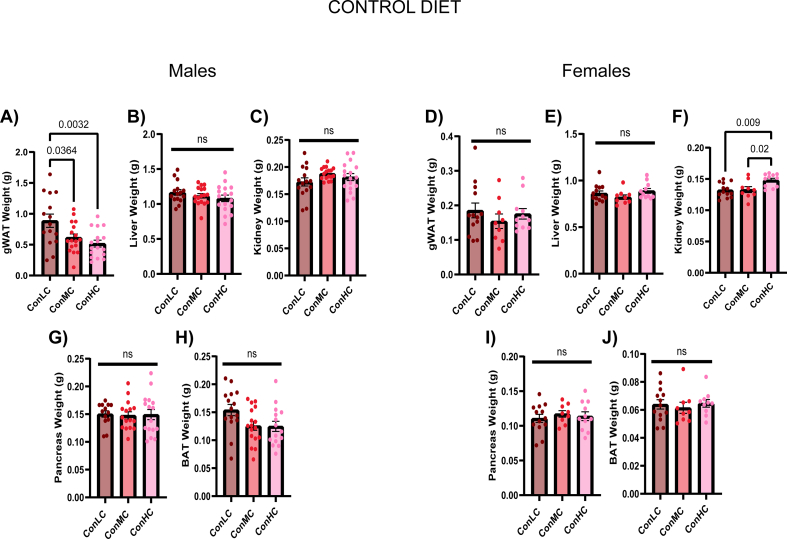
**Low choline intake increases gonadal white adipose tissue weight in male mice under nonobese conditions.** Tissue weights in males (**A-C, G, H**) and females (**D-F, I, J**). Gonadal white adipose tissue (gWAT) weight in males (**A**) and females (**D**), liver weight in males (**B**) and females (**E**), kidney weight in males (**C**) and females (**F**), pancreas weight in males (**G**) and females (**I**), and brown adipose tissue (BAT) weight in males (**H**) and females (**J**). Sample sizes per group: ConLC (males n = 15, females n = 13), ConMC (males n = 17, females n = 9), and ConHC (males n = 17, females n = 11). A one-way ANOVA followed by a Tukey's multiple comparisons test was used for (**A**), (**B**), (**F**), (**H**), and (**I**). A Kruskal–Wallis one-way ANOVA with Dunn's multiple comparisons test was used for (**G**). A Welch one-way ANOVA with a Dunnett T3 multiple comparisons test was used for (**C**). A lognormal one-way ANOVA with a Tukey multiple comparisons test was used for (**D**), (**E**), and (**J**). Statistical significance was set at p < 0.05. Data are presented as mean ± SEM, with individual data points representing single animals.

To localize the body composition changes, we weighed individual tissues at sacrifice (Suppl. Fig. 2). In males, gonadal white adipose tissue (gWAT) was significantly heavier in ConLC than in ConMC (**p** = **0.0364**) and ConHC (**p** = **0.0032**) (Suppl. Fig. 2A). Liver, kidney, pancreas and BAT weights did not differ across choline groups (Suppl. Figs. 2B, C, G, H).

In females, gWAT, liver, pancreas, and BAT weights were unchanged across choline groups (Suppl. Figs. 2D, E, I, J). The only difference was kidney weight, which was higher in ConHC than in ConLC (**p** = **0.009**) and ConMC (**p** = **0.02**) (Suppl. Fig. 2F). This isolated change was not accompanied by differences in body weight, fat or lean mass that would account for it.

### 3.4 Sustained low choline intake disrupts systemic metabolic signaling in male mice

**Figure 2:**
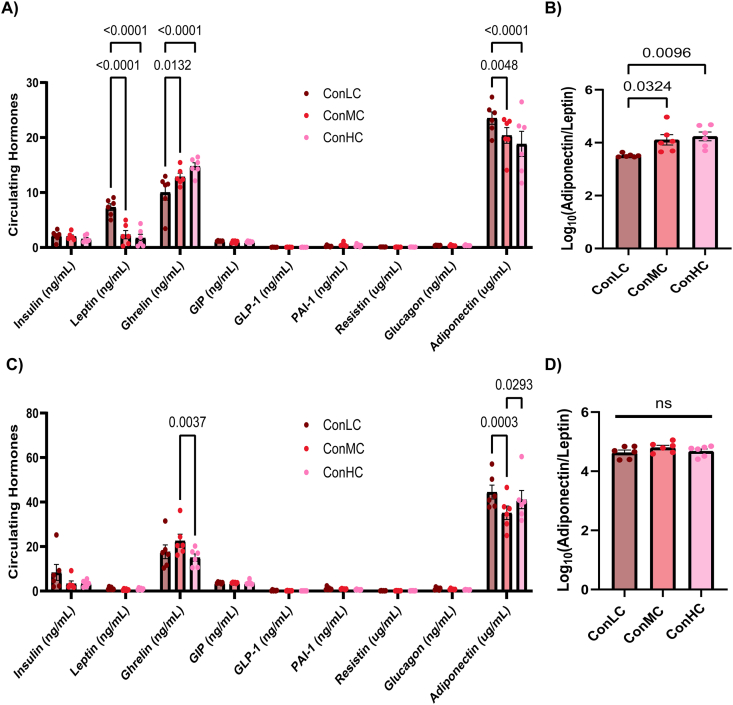
**Low choline intake alters circulating endocrine profiles and increases leptin in males under nonobese conditions.** Circulating hormones in males (**A**) and females (**C**), and log10-transformed adiponectin-to-leptin ratios (**B, D**) across low (LC), medium (MC), and high (HC) choline intake groups. Sample sizes per group: ConLC (males *n* = 6, females *n* = 6), ConMC (males *n* = 6, females *n* = 6), and ConHC (males *n* = 6, females *n* = 6). A two-way ANOVA followed by a Tukey multiple comparisons test was performed for (**A**) and (**C**). A one-way ANOVA followed by a Tukey multiple comparisons test was performed for (**B**) and (**D**). Statistical significance was set at *p* < 0.05. Data are presented as mean ± SEM, with individual data points representing single animals.

We next interrogated the circulating hormonal environment to determine if sustained low choline intake is accompanied by systemic endocrine remodeling (Figure 2). Males consuming ConLC exhibited significantly elevated circulating leptin (**p**< **0.0001 vs ConMC and ConHC**), along with reduced ghrelin relative to ConMC and ConHC (**p** < **0.0132** and **0.0001**, respectively) (Figure 2A). Adiponectin concentrations were also increased in ConLC males compared to ConMC (**p = 0.0048**) and HC (**p** < **0.0001**) (Figure 2A). Despite elevated adiponectin, the adiponectin to leptin ratio was significantly reduced in ConLC males relative to ConMC (**p** = **0.0324**) and ConHC **(p = 0.0096**) (Figure 2B).

In contrast, females did not exhibit choline-dependent differences in circulating leptin (Figure 2C). Ghrelin concentrations were increased in ConMC relative to ConHC (**p = 0.0037**) (Figure 2C). Adiponectin was elevated in ConLC females compared to ConMC (**p = 0.0003**) and ConHC (**p = 0.0293**) (Fig 2C). However, the adiponectin-to-leptin ratio did not differ significantly across choline groups (Figure 2D).

These findings demonstrate that sustained low choline intake under control diet conditions is associated with a coordinated shift in circulating metabolic signals in males. However, in females these signals are limited and do not extend to integrated hormonal indices such the adiponectin-to-leptin ratio.

### 3.5 High-fat feeding masks the metabolic impact of low dietary choline

Given the impact of low dietary choline intake on metabolic health under a control diet, we next asked if these effects persist under diet-induced obesity across graded levels of choline availability. Using different choline intakes, 79C57BL/6J mice (49 males, 30 females) were randomized to receive either a high-fat (HF) diet containing low (HFLC; males n = 17, females n = 10), medium (HFMC; males n = 17, females n = 10), or high (HFHC; males n = 15, females n = 10) choline for 15 weeks (Suppl. Fig. 1A).

**Figure 3:**
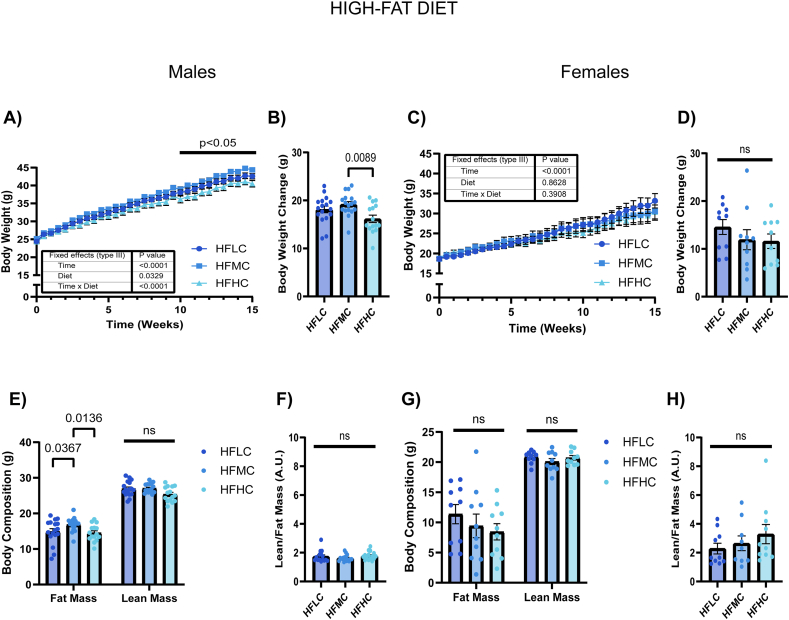
**High-fat feeding masks choline-dependent effects on body composition and metabolic phenotype.** Body weight over time with corresponding mixed-effects models in males (**A**) and females (**C**), and body weight change from baseline to study endpoint (**B, D**) in mice fed a high-fat diet with low (LC), medium (MC), and high (HC) choline intake. Body composition (fat mass and lean mass) in males (**E**) and females (**G**), and lean-to-fat mass ratio in males (**F**) and females (**H**). HFLC (males *n* = 17, females *n* = 10), HFMC (males *n* = 17, females *n* = 10), and HFHC (males *n* = 15, females *n* = 10). A Mixed-effects analysis followed by a Tukey multiple comparisons test was performed for (**A**) and (**C**). A two-way ANOVA followed by a Tukey multiple comparisons test was performed for (**E**) and (**G**). A one-way ANOVA followed by a Tukey multiple comparisons test was used for (**B**) and (**D**). A Kruskal–Wallis one-way ANOVA with Dunn's multiple comparisons test was used for (**F**) and a lognormal one-way ANOVA followed by a Tukey multiple comparisons test was performed for (**H**). Statistical significance was set at *p* < 0.05. Data are presented as mean ± SEM, with individual data points representing single animals.

**Supplementary Figure 3:**
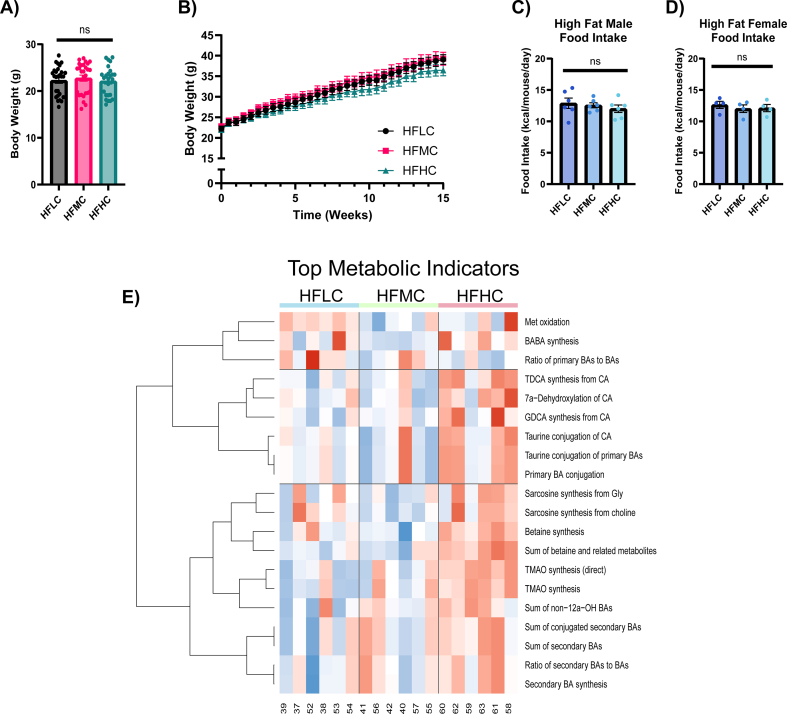
**High-fat feeding limits the resolution of choline-dependent body weight effects.** Baseline body weight (**A**) and body weight trajectory over time (**B**) in males and females fed a high-fat diet. Male (**C**) and female (**D**) food intake. (**E**) Unsupervised hierarchical clustering of top altered metabolic indicators in male livers across choline intake groups. For (**A**) and (**B**), sample sizes per group: HFLC (males n = 17, females n = 10), HFMC (males n = 17, females n = 10), and HFHC (males n = 15, females n = 10). For (**C**) and (**D**), food intake is averaged per cage (total cages n = 18 male and n = 12 female), where each point represents a cage with 2–5 mice. For (**E**), sample sizes per group: HFLC (males n = 6), HFMC (males n = 6), and HFHC (males n = 6). A Kruskal–Wallis one-way ANOVA with Dunn's multiple comparisons test was used for (**A**) while a one-way ANOVA followed by a Tukey's multiple comparisons test was used for (**C**) and (**D**). A Mixed-effects analysis followed by a Tukey multiple comparisons test was used for (**B**). Statistical significance was set at p < 0.05. Data are presented as mean ± SEM, with individual data points representing single animals.

When analyzed across sexes, baseline body weight and longitudinal body weight trajectories did not differ among dietary groups (Suppl. Fig. 3A and B), while when analyzed separately by sex, males exhibited weight gain over time, with a modest effect of diet (**p = 0.0329**) and a significant time × diet interaction (**p** < **0.0001**) by mixed-effects modeling (Figure 3A). Among choline groups, body weight change from baseline to study end differed only between HFMC and HFHC, with reduced weight gain in HFHC (**p = 0.0089**); the HFLC group did not differ from the others (Figure 3B). In contrast, females consuming the HF diet demonstrated significant weigh gain over time (**p** < **0.0001**) with no effect of dietary choline and no time × diet interaction (Figure 3C,D). Food intake did not differ across the HF diet choline groups, indicating that body weight differences were not driven by caloric intake, and profiling of hepatic choline-related metabolites in a representative subset of high-fat-fed males confirmed effective choline delivery, with canonical choline-derived metabolites such as TMAO and betaine trending lower in HFLC and higher in HFHC (Suppl. Fig. 3C–E).

### 3.6 Choline intake has limited effects on body composition under a high-fat diet

Fat mass increased across all HF diet groups, consistent with diet-induced obesity. In males, graded choline intake modestly altered fat mass, HFMC mice had greater fat mass than both HFLC (**p** = **0.0367**) and HFHC (**p** = **0.0136**), whereas lean mass and the lean-to-fat ratio did not differ across choline groups (Figure 3E,F). In females, fat mass, lean mass, and the lean-to-fat mass ratio were unchanged across choline groups (Figure 3G,H).

### 3.7 Choline intake has limited effects on tissue weights and circulating hormones under a high-fat diet

**Supplementary Figure 4:**
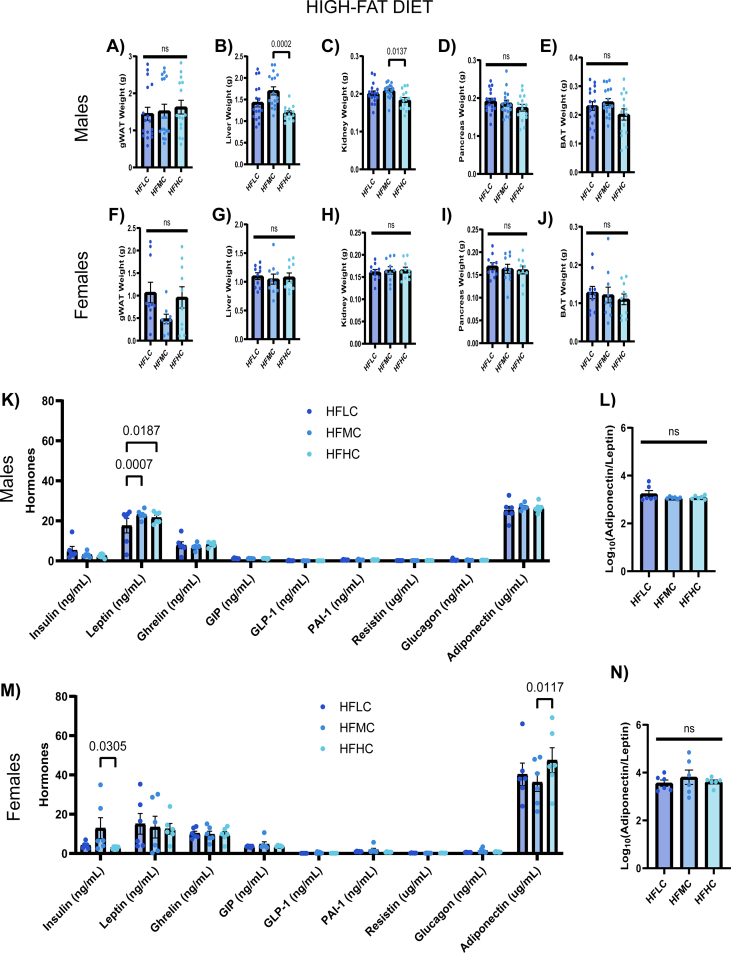
**High-fat feeding masks the choline-dependent effects on metabolic effects.** Tissue weights in high-fat-diet males (**A-E**) and females (**F-J**). Gonadal white adipose tissue (gWAT) weight in males (**A**) and females (**F**), liver weight in males (**B**) and females (**G**), kidney weight in males (**C**) and females (**H**), pancreas weight in males (**D**) and females (**I**), and brown adipose tissue (BAT) weight in males (**E**) and females (**J**). Circulating hormones in males (**K**) and females (**M**), and adiponectin-to-leptin ratios in males (**L**) and females (**N**). For (**A-J**) sample sizes per group: HFLC (males n = 17, females n = 10), HFMC (males n = 17, females n = 10), and HFHC (males n = 15, females n = 10). For (**K–N**), Sample sizes per group: HFLC (males n = 6, females n = 6), HFMC (males n = 6, females n = 6), and HFHC (males n = 6, females n = 6). A one-way ANOVA followed by a Tukey's multiple comparisons test was used for (**C**), (**G**), (**H**), (**D**), (**E**), (**I**), and (**J**). A Kruskal–Wallis test followed by a Dunn's multiple comparisons test was used for (**A**). A Welch one-way ANOVA test followed by a Dunnett T3 multiple comparisons test was used for (**B**) while a lognormal one-way ANOVA was performed followed by a Tukey's multiple comparisons for (**F**). A two-way ANOVA followed by a Tukey multiple comparisons test was used for (**K**) and (**M**). A Kruskal–Wallis test followed by a Dunn's multiple comparisons test was used for (**L**) while a Welch one-way ANOVA test followed by a Dunnett T3 multiple comparisons test was used for (**N**). Statistical significance was set at p < 0.05. Data are presented as mean ± SEM, with individual data points representing single animals.

We weighed individual tissues at sacrifice in the HF cohort (Suppl. Fig. 4). In males, gWAT, pancreas and BAT weights did not differ across choline groups (Suppl. Fig. 4A, D and E). Liver and kidney weights were lower in HFHC than in HFMC (**p** = **0.0002** and **p** = **0.0137**, respectively) (Suppl. Fig. 4B and C). In females, gWAT, liver, kidney, pancreas, and BAT weights did not differ across choline groups. (Suppl. Fig. 4F–J).

Likewise, we observed limited differences in circulating hormones across choline concentrations. In males, leptin was lower in HFLC than in HFMC (**p** = **0.0007**) and HFHC (**p** = **0.0187**), while in females, insulin was lower in HFHC than in HFMC (**p** = **0.0305**), and adiponectin was higher in HFHC than in HFMC (**p** = **0.0117**) (Suppl. Fig. 4K **and M**). However, these changes did not translate into differences in integrated hormonal indices, as adiponectin-to-leptin ratios were not altered across groups (Suppl. Fig. 4L **and N**).

Our results indicate that under conditions of diet-induced obesity, the metabolic effects of graded choline intake are largely attenuated, suggesting that excess caloric intake overrides choline-dependent metabolic regulation.

### 3.8 Sustained low choline intake induces steatohepatitis in male but not female mice

**Figure 4:**
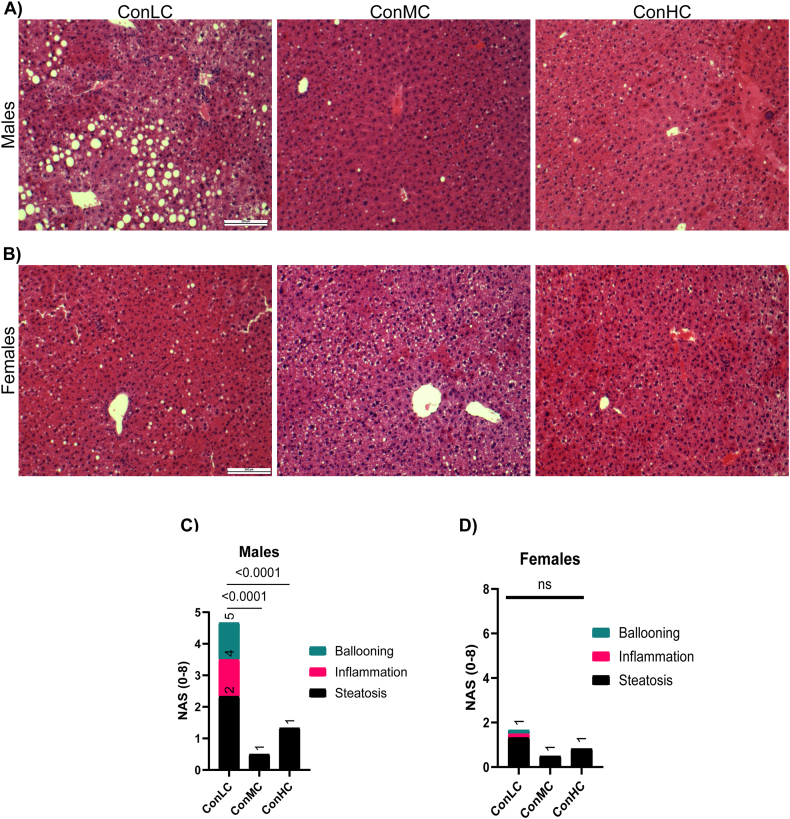
**Low choline intake induces steatotic liver pathology in male (but not female) mice under nonobese conditions.** (**A**) Representative hematoxylin and eosin (H&E)-stained liver sections from males and females (**B**) across low (LC), medium (MC), and high (HC) choline intake groups, with corresponding NAFLD activity scores (NAS) in males (**C**) and females (**D**). Sample sizes per group: ConLC (males n = 6, females n = 6), ConMC (males n = 6, females n = 6), and ConHC (males n = 6, females n = 6). A two-way ANOVA followed by a Tukey's multiple comparisons test was used for (**C**) and (**D**). Statistical significance was set at p < 0.05.

Given the established role of the liver as the primary organ sensitive to choline availability, and the canonical site of injury in choline-deprivation models [36], we next examined hepatic pathology. Males consuming ConLC exhibited marked macrovesicular steatosis relative to ConMC and ConHC (Figure 4A). Blinded histopathological scoring revealed a NAFLD Activity Score (NAS) of 5 in ConLC males, driven by increased steatosis, inflammation, and hepatocellular ballooning (Figure 4C). This score was significantly higher than ConMC and ConHC (**p** < **0.0001**), indicating that sustained low choline intake induces steatohepatitis-like pathology in males under non-obesogenic conditions.

In contrast, females exhibited minimal histopathological changes (Figure 4B), with NAS values near 1–2, with no significant differences across choline levels (Figure 4D). Steatosis, inflammation, and ballooning were limited across groups of female mice, indicating that hepatic injury under control diet conditions was selectively observed in males.

**Supplementary Figure 5:**
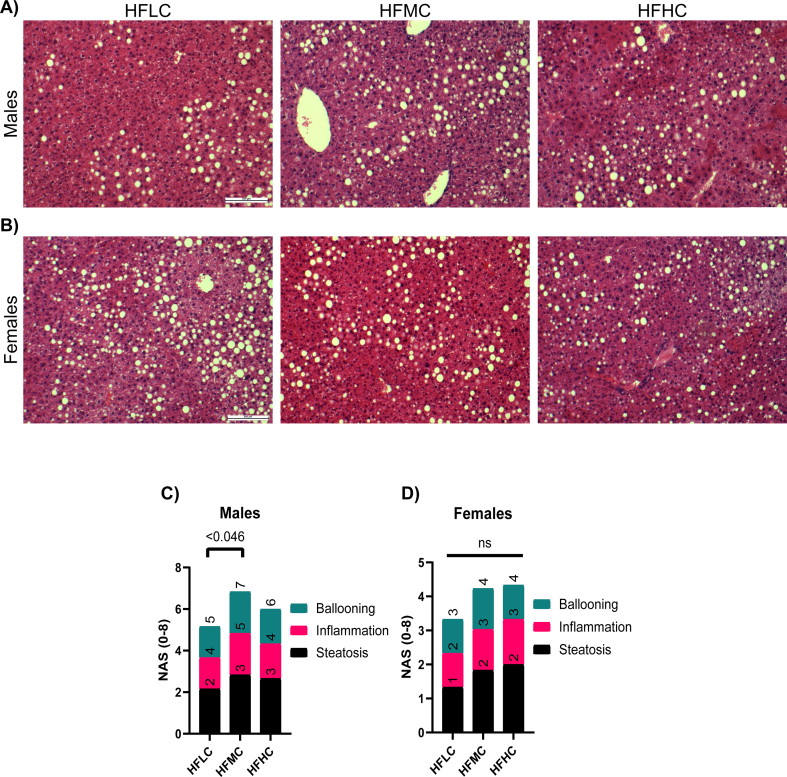
**High-fat feeding induces hepatic steatosis independent of dietary choline intake.** Representative hematoxylin and eosin (H&E)-stained liver sections from males (**A**) and females (**B**), with corresponding NAFLD activity scores (NAS) in males (**C**) and females (**D**). Sample sizes per group: HFLC (males n = 6, females n = 6), HFMC (males n = 6, females n = 6), and HFHC (males n = 6, females n = 6). A two-way ANOVA followed by a Tukey's multiple comparisons test was used for (**C**) and (**D**). Statistical significance was set at p < 0.05.

In the HF diet context, diffuse macrovesicular steatosis across all choline groups were observed in both males and females, with no clear qualitative differences in lipid droplet accumulation between HFLC, HFMC and HFHC (Suppl. Fig. 5 **A and B**). Blinded histopathological scoring showed NAS values consistently elevated across all HF groups, ranging from 5 to 7 in males and 3–4 in females (Suppl. Fig. 5C and D). In males, a modest difference was detected between HFLC and HFMC (**p** < **0.046**); however, this did not follow graded pattern across choline levels. In females, NAS scores did not differ significantly across choline groups. These data indicate HF feeding induces hepatic pathology independent of choline availability, with no consistent choline-dependent effects on steatosis, inflammation or ballooning under these conditions.

### 3.9 Low choline intake remodels hepatic metabolism and triglyceride architecture without increasing total TAG mass

Given that the dominant and reproducible signal occurs in males under control diet with low choline exposure, subsequent analyses were restricted to this condition to define the underlying mechanisms.

**Figure 5:**
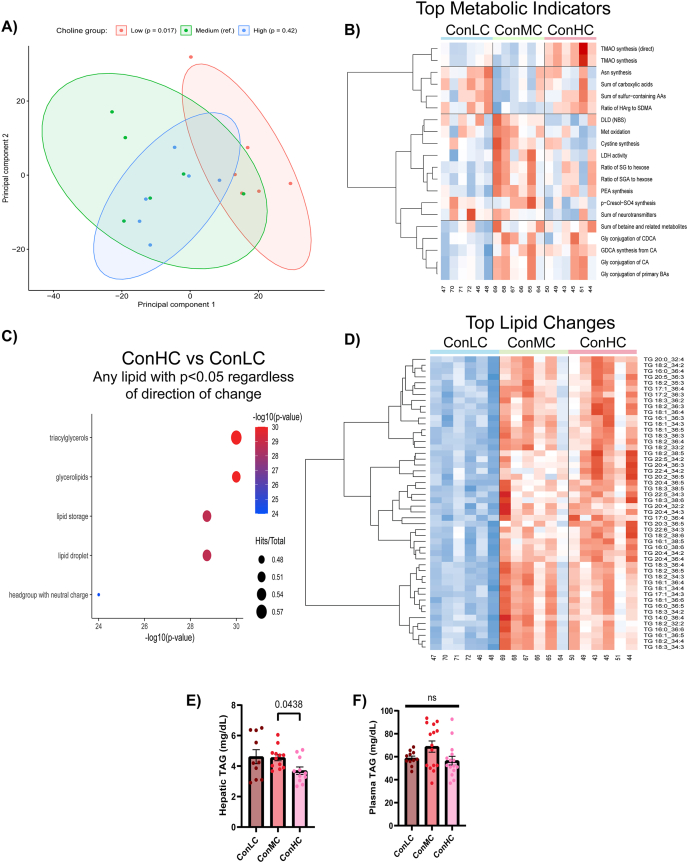
**Low choline intake in male mice remodels hepatic metabolism and triglyceride architecture without increasing total TAG content.** Principal component analysis (PCA) of hepatic metabolomic profiles (**A**) and unsupervised hierarchical clustering of top altered metabolic indicators (**B**) in male livers across low (LC), medium (MC), and high (HC) choline intake groups. (**C**) LION enrichment analysis highlighting lipid ontology terms associated with triglyceride and lipid storage pathways. (**D**) Heatmap of top altered triglyceride (TAG) species across acyl chain length and degree of unsaturation. Total hepatic TAG content (**E**) and plasma TAG concentrations (**F**). For (**A–D**), sample sizes per group: ConLC (n = 6), ConMC (n = 5), and ConHC (n = 6); for (**E)** sample sizes per group: ConLC (n = 10), ConMC (n = 13), and ConHC (n = 12); for **(F**) sample sizes per group: ConLC (n = 12), ConMC (n = 15), and ConHC (n = 15). A Welch's one-way ANOVA followed by a Dunnett's T3 multiple comparisons test was used for (**E**) and (**F**). Statistical significance was set at p < 0.05. Data are presented as mean ± SEM, with individual data points representing single animals.

To define the hepatic metabolic consequences of graded choline intake under control diet conditions, we performed targeted metabolomic profiling (Figure 5). Principal component analysis revealed clear separation of ConLC from ConMC and ConHC (**p** = **0.017**), indicating a dominant metabolic shift driven by low choline availability (Figure 5A).

The non-lipid metabolic indicators with the most significant group differences further demonstrated that dietary choline intake is directly reflected in the hepatic metabolome (Figure 5B). Canonical choline-derived metabolites, including TMAO and betaine, trended lower in ConLC and higher in ConHC, confirming effective modulation of choline dependent pathways. In parallel, metabolites related to one-carbon metabolism, sulfur amino acid flux, and glycine-conjugated bile acids showed coordinated trends, including reductions in primary bile acid conjugation and betaine-related intermediates, indicating that low choline availability propagates beyond substrate limitation to reshape interconnected metabolic pathways.

To further contextualize these changes, LION enrichment analysis (ConLC vs ConHC) identified strong enrichment of lipid ontology terms related to triacylglycerols, glycerolipids, lipid storage, and lipid droplets, supporting a coordinated shift in lipid handling pathways (Figure 5C). We next interrogated hepatic lipid composition under graded choline intake. Lipidomic profiling revealed clear segregation of ConLC from ConMC and ConHC mice, driven by broad remodeling of triglyceride (TAG) species across acyl chain length and degree of unsaturation (Figure 5D).

To determine whether these compositional shifts translated into altered bulk triglyceride storage, total hepatic TAG was quantified using a colorimetric assay independent of acyl composition. Total hepatic TAG content did not increase in ConLC mice; instead, a modest reduction was observed in ConHC relative to ConMC (**p = 0.0438**) (Figure 5E). Plasma TAG concentrations likewise did not differ among choline groups (Figure 5F). These findings demonstrate that low choline does not increase total hepatic lipid burden but instead drives a reorganization of triglyceride composition and lipid storage architecture, consistent with a shift in hepatic handling rather than simple lipid accumulation.

### 3.10 Low choline intake induces transcriptional remodeling of hepatic lipid metabolism

**Figure 6:**
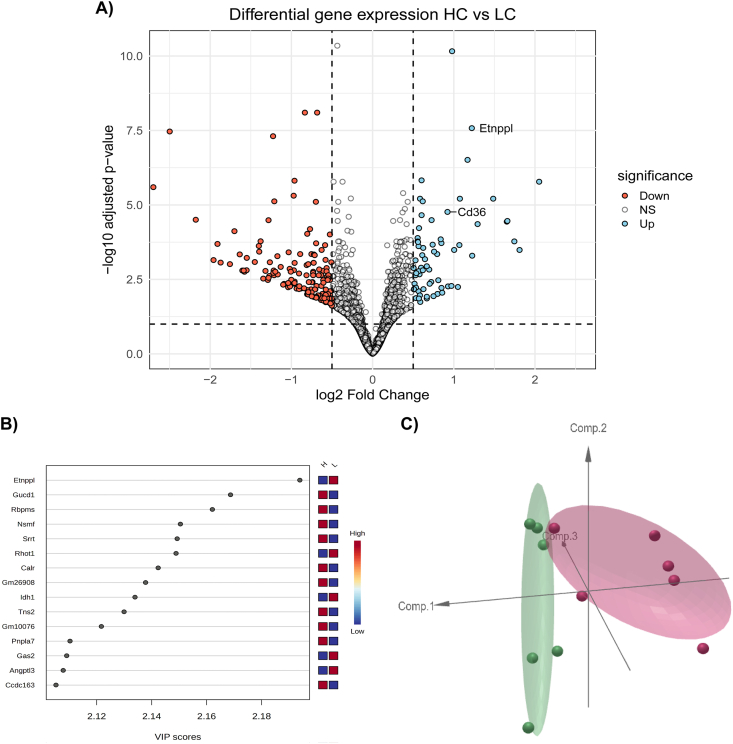
**Low choline intake in male mice induces a distinct hepatic transcriptional program highlighted by increased Etnppl expression.** (**A**) Volcano plot of differential gene expression comparing male ConLC and ConHC livers. (**B**) Top transcripts separating ConLC and ConHC groups based on variable importance in projection (VIP) scores. (**C**) Multiple co-inertia analysis demonstrating separation between ConLC (green) and ConHC (red) transcriptomic profiles. Sample sizes per group: ConLC (n = 6) and ConHC (n = 6). Statistical significance was set at p < 0.05.

Since we observed extensive lipid remodeling in response to ConLC intake, with the most pronounced differences occurring between ConHC and ConLC, we next examined if these alterations were reflected at the transcriptional level. Consistent with our metabolomic and lipidomic analyses, the dominant separation across conditions was driven by the ConLC group, indicating that low choline intake defines the primary axis of metabolic variation. Transcriptomic profiling revealed significant differential expression of genes when comparing ConHC and ConLC mouse liver (Figure 6A), including transcripts related to lipid and phospholipid metabolism. The directionality of these changes mirrored the metabolic phenotype, supporting coordinated regulation across molecular layers.

Partial least squares discriminant analysis (PLS-DA) further identified a set of transcripts driving the separation between dietary groups, among which ethanolamine-phosphate phospho-lyase (*Etnppl*) emerged as top discriminant feature, positioning it as a candidate regulator linking choline availability to phospholipid and lipid metabolic remodeling (Figure 6B). To further integrate these datasets, we performed Multiple Co-Inertia Analysis (MCIA) across lipidomic, metabolomic, and transcriptomic layers. This analysis revealed a clear separation between ConLC and ConHC samples, with lipidomics features contributing most strongly to the variance explaining group separation (Figure 6C), indicating lipid remodeling represents the dominant molecular signature of the choline-dependent phenotype.

### 3.11 Low choline intake engages a Cd36-ETNPPL axis linked to insulin resistance

**Figure 7:**
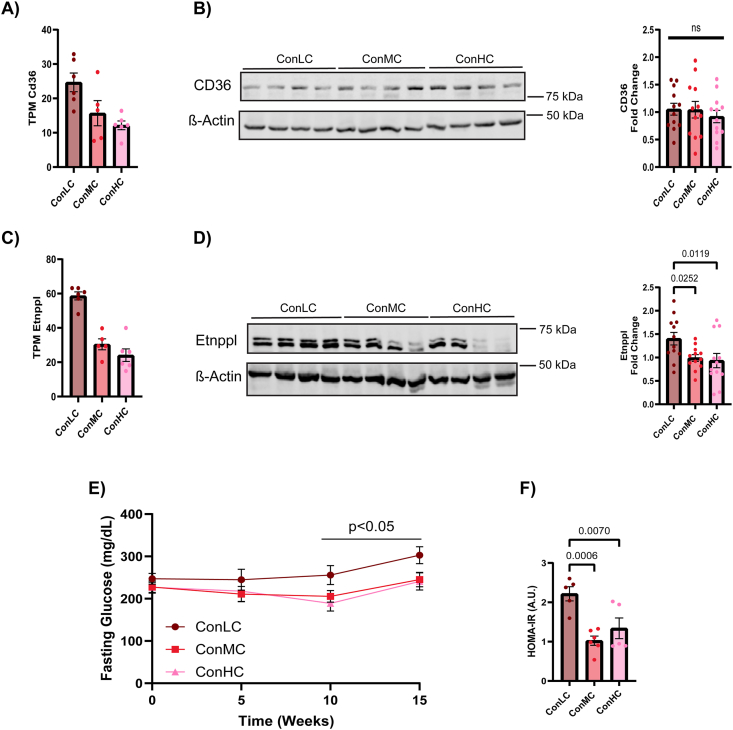
**Low choline intake in male mice increases *Etnppl* expression and promotes metabolic dysfunction.** Cd36 transcript abundance (TPM) (**A**) and protein expression (**B**) in male liver. *Etnppl* transcript abundance (TPM) (**C**) and protein expression (**D**) in male liver. TPM values are shown for visualization, and statistical significance was determined from differential expression analysis of raw counts. Male fasting glucose over time (**E**) and HOMA-IR (**F**). For (**A**) and (**C**), sample sizes per group: ConLC (n = 6), ConMC (n = 5), and ConHC (n = 6) For (**B**) and (**D**), sample sizes per group: ConLC (n = 12), ConMC (n = 12), and ConHC (n = 12). For (**E**), sample sizes per group: ConLC (n = 15), ConMC (n = 17), and ConHC (n = 17). For (**F**), sample sizes per group: ConLC (n = 6), ConMC (n = 6), and ConHC (n = 5). A one-way ANOVA followed by a Fisher's LSD multiple comparisons test was used for (**B**) and (**D**). A two-way ANOVA followed by a Fisher's LSD multiple comparisons test was performed for (**E**). A one-way ANOVA followed by a Fisher's LSD multiple comparisons test was used for (**F**). Statistical significance was set at p < 0.05. Data are presented as mean ± SEM, with individual data points representing single animals.

Given the coordinated remodeling of hepatic lipid species and the emergence of *Etnppl* as a top transcriptional response to low choline intake, we next asked if this metabolic state is accompanied by activation of lipid sensing pathways. Among candidate lipid sensors, CD36 is well established as a key mediator of dietary lipid sensing, and it is positioned as a central node in metabolic adaptation to lipid flux. Our transcriptomic analysis revealed that *Cd3*6 mRNA was upregulated in livers from ConLC-treated mice, and its expression changed with dietary choline intake in a monotonic manner (Figure 7A). However, when we examined CD36 protein abundance by Western blot, we did not observe significant differences among dietary groups (Figure 7B). Although protein levels were unchanged, this finding does not exclude alterations in lipid sensing activity.

*Etnppl* mRNA also responded to dietary choline intake in a dose-dependent manner (Figure 7C). We therefore examined ETNPPL protein levels and found that hepatic abundance was significantly increased in ConLC males relative to ConMC and ConHC (**p** = **0.0252** and **p** = **0.0119**, respectively) (Figure 7D), confirming that transcriptional induction translates to increased enzyme abundance under low choline conditions.

Because both CD36-mediated lipid sensing and Etnppl-dependent phospholipid metabolism have been linked to insulin resistance, we evaluated systemic metabolic outcomes. Circulating glucose rose over time in ConLC mice and was significantly elevated relative to ConMC and ConHC after 15 weeks (**p < 0.05**) (Figure 7E). Using circulating insulin concentrations from the hormone panel (Fig 2A), we calculated the Homeostatic Model Assessment for Insulin Resistance (HOMA-IR) as an index of insulin sensitivity. Consistent with the glucose data, ConLC males showed significantly elevated HOMA-IR relative to ConMC and ConHC (**p** = **0.0006** and **p** = **0.0070**, respectively) (Figure 7F), demonstrating the development of insulin resistance under non-obesogenic conditions.

**Supplementary Figure 6:**
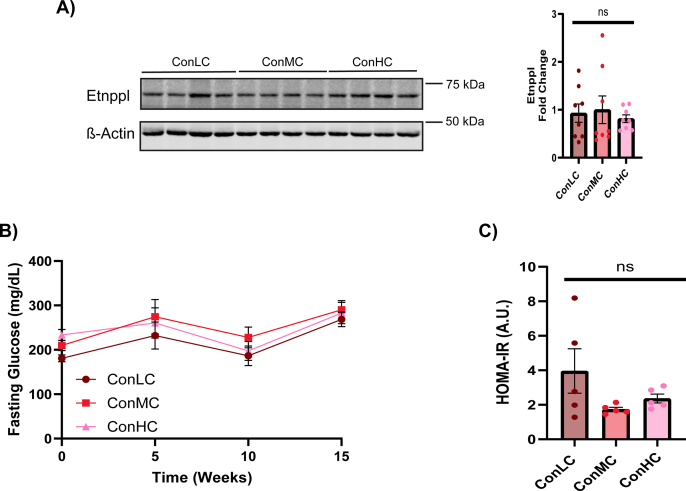
**Neither glucose homeostasis nor hepatic Etnppl protein expression is altered by dietary choline intake in female mice.** Etnppl protein expression (**A**) in female liver. Female fasting glucose over time (**B**) and HOMA-IR (**C**). For (**A**), sample sizes per group: ConLC (n = 8), ConMC (n = 8), ConHC (n = 8). For (**B**), sample sizes per group: ConLC (n = 13), ConMC (n = 9), ConHC (n = 11). For (**C**), sample sizes per group: ConLC (n = 6), ConMC (n = 5), and ConHC (n = 6). A lognormal one-way ANOVA was performed followed by a Tukey's multiple comparisons for (**A**). A two-way ANOVA followed by a Fisher's LSD multiple comparisons test was performed for (**B**). A one-way ANOVA followed by a Tukey multiple comparisons test was used for (**C**). Statistical significance was set at p < 0.05. Data are presented as mean ± SEM, with individual data points representing single animals.

**Supplementary Figure 7:**
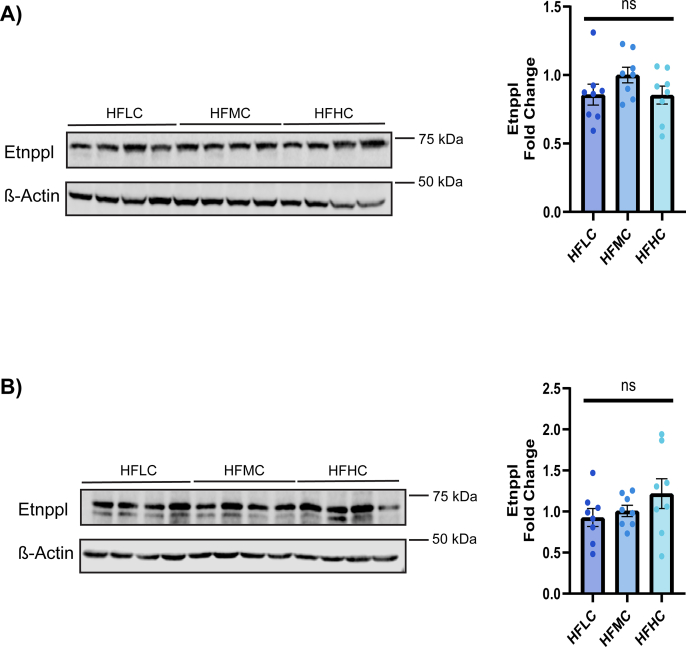
**Hepatic Etnppl protein expression is not altered by dietary choline intake in either males or females fed a high-fat diet.** Hepatic Etnppl protein quantification in males (**A**) and females (**B**). For (**A-B**), sample sizes per group: HFLC (n = 8), HFMC (n = 8), and HFHC (n = 8). A one-way ANOVA followed by a Tukey's multiple comparisons test was used for both (**A**) and (**B**). Statistical significance was set at p < 0.05. Data are presented as mean ± SEM, with individual data points representing single animals.

This phenotype was confined to low-choline males on the Con conditions. In females, hepatic ETNPPL protein, circulating glucose, and HOMA-IR did not differ across choline groups (Suppl. Fig. 6A–C). Under HF feeding, hepatic ETNPPL did not differ across choline groups in either sex (Suppl. Fig. 7A and B).

Hepatic ETNPPL induction, and the insulin resistance that accompanied it, were therefore specific to choline-restricted males on the control diet.

## 4 Discussion

The present study identifies sustained low dietary choline intake as a driver of hepatic metabolic dysfunction, linking micronutrient availability to lipid remodeling and insulin resistance. A central finding is the induction of *Etnppl* under low choline conditions, positioning this enzyme as a potential mediator of choline-dependent metabolic adaptation. The metabolic phenotype emerged first at the whole-body level. Male mice consuming low choline gained more weight and exhibited altered body composition, whereas females were largely protected, consistent with known sex differences in choline metabolism [37]. This divergence likely reflects estrogen-dependent regulation of phosphatidylethanolamine N-methyl transferase (Pemt), which supports endogenous PtdCho synthesis and may buffer females against reduced dietary choline availability [38].

These systemic changes were accompanied by a coordinated endocrine response in male mice. Elevated leptin, increased adiponectin, reduced ghrelin, and a decreased adiponectin-to-leptin ratio indicate a metabolically dysregulated state associated with chronic nutritional stress [[39], [40], [41]]. Consistent with its established role as a proxy of metabolic health [42,43], the reduced adiponectin-to-leptin ratio in ConLC males indicates impaired metabolic status. Ghrelin concentrations were concurrently reduced, a pattern reported in obesity [43,44]. Together, this endocrine profile suggests that low choline intake in a nonobese context promotes systemic metabolic dysfunction. In contrast, under HF diet-induced obesity conditions, graded choline intake had minimal impact on metabolic outcomes. This likely reflects dominance of the obesogenic phenotype, which masks choline-specific effects. Differences between our findings and a recent study [45] are likely attributable to the supraphysiological choline doses used in those models. Specifically, ∼17 g/kg was used in their study whereas our study used 6.3 g/kg, and our dietary design was not intended to test pharmacologic choline supplementation but rather to model physiologically relevant intake ranges based on current human intake estimates. However, these differences in design reflect ongoing discussion in the field regarding the need for harmonization of dietary models to facilitate comparisons across studies and improve translation [46].

Consistent with systemic findings, histological analyses revealed that male mice consuming a ConLC diet developed metabolic dysfunction-associated steatotic liver disease (MASLD), with NAS scores comparable to those observed under HF feeding conditions. In contrast, females displayed milder hepatic changes under the same dietary conditions. These findings align with the well-established observation that inadequate choline availability disrupts hepatic lipid homeostasis and promotes liver injury [34,47]. Importantly, the phenotype observed here differs from the classical methionine-choline deficient (MCD) dietary model commonly used to induce experimental steatohepatitis, which does not exhibit insulin resistance [48]. Methionine concentrations were equal across all our diets and sufficient to meet physiological requirements, indicating that the observed phenotype arises from sustained low choline intake under otherwise nutritionally complete conditions. Unlike classical choline-deprivation models, which induce rapid steatosis through complete removal of dietary choline, our data demonstrate that sustained low choline intake is sufficient to produce progressive hepatic pathology in males. The similarity between the ConLC and HF phenotypes further suggests that chronic choline insufficiency may predispose the liver to metabolic stress independent of excess caloric intake.

Our data suggest that the metabolic impact of low choline intake is driven by lipid remodeling rather than lipid accumulation. Lipid composition and spatial organization are increasingly recognized as key determinants of cellular function, with alterations in lipid species capable of reshaping signaling, membrane dynamics, and metabolic flux independent of bulk lipid accumulation [49,50]. In this context, the coordinated remodeling of triglyceride species indicates a shift in lipid utilization pathways but does not necessarily reflect increased oxidation alone. An alternative, non-mutually exclusive explanation is that low choline availability induces oxidative stress [51], leading to selective modification or turnover of triglyceride species. Consistent with this, transcriptional changes observed here and discussed below may increase production of reactive intermediates such as acetaldehyde and ammonia, which can promote lipid oxidation and preferentially impact triglyceride composition.

This interpretation is further supported by the tight coupling between phospholipid metabolism and lipid handling. Choline availability directly constraints PtdCho synthesis, a central component of membrane integrity and lipid droplet dynamics [52]. Disruption of this axis is expected to alter lipid trafficking, favoring redistribution and metabolic utilization of triglycerides without increasing total lipid storage. Although remodeling was observed across multiple lipid classes, including fatty acids, sphingolipids and phospholipids, the most robust, significant and consistent changes occurred in triglyceride species. The apparent selectivity toward triglyceride remodeling, rather than global changes across all lipid classes, may reflect their central role as a dynamic buffer for fatty acids under conditions of metabolic and oxidative stress. This type of lipid remodeling, where changes in lipid species occur without parallel changes in total lipid content, is consistent with systems in which lipid turnover and utilization are dynamically regulated [53,54]. Importantly, this remodeling precedes overt changes in total triglyceride content and may represent an early adaptive response that maintains lipid balance through increased utilization, or selective remodeling under stress rather than accumulation.

Transcriptomic analysis identified *Etnppl* as the top choline-responsive gene, linking phospholipid metabolism to the observed lipid phenotype. ETNPPL catalyzes the degradation of phospho-ethanolamine (p-Etn), a Kennedy-pathway intermediate, to acetaldehyde, ammonia, and inorganic phosphate; by depleting p-Etn, it constraints the precursor pool available for phosphatidylethanolamine (PtdEtn) synthesis and thereby shapes membrane phospholipid composition. In the context of an altered PtdCho:PtdEtn ratio, increased *Etnppl* expression and protein levels may reflect a compensatory response to reduced choline, and thus reduced PtdCho synthesis, acting through depletion of p-Etn to modulate PtdEtn availability and maintain phospholipid homeostasis. Alterations in this ratio are widely recognized as indicators of membrane stress and metabolic dysfunction, particularly in MASLD [55,56]. Beyond membrane structure, changes in PtdEtn availability have important consequences for mitochondrial function [[57], [58], [59]]. PtdEtn synthesis through the Kennedy pathway contributes to mitochondrial membrane composition and supports oxidative metabolism [60]. Disruption of this axis therefore provides a mechanistic link between altered phospholipid homeostasis and impaired mitochondrial function. Consistent with this, forced ETNPPL expression in hepatoma cells raises the PtdCho:PtdEtn ratio and lowers mitochondrial ATP production and oxygen consumption [61], indicating that elevated ETNPPL activity is sufficient to shift phospholipid balance and depress oxidative metabolism. In this context, phospholipid imbalance may represent an upstream event driving the broader lipid remodeling observed under sustained low choline intake. Importantly, this regulatory role appears to be context dependent. *Etnppl* knockout models exhibit relatively mild metabolic phenotypes, suggesting that ETNPPL activity becomes metabolically relevant primarily under conditions of nutrient stress [62], with more pronounced effects observed in *Etnppl* knockout female mice [63]. Gain-of function studies further connect ETNPPL to the functional endpoint we observed, in hepatocyte models, elevated ETNPPL promotes hepatic insulin resistance through ROS-dependent signaling, impairing autophagy via the ARG2-ROS axis [64]. Our findings expand this concept by identifying ETNPPL as a choline-responsive enzyme whose expression increases under sustained choline insufficiency. Critically, this induction was not a generalized response to choline intake. Hepatic ETNPPL protein did not differ across choline levels in females, parallelling their lack of hyperglycemia and insulin resistance and consistent with estrogen-dependent PEMT activity preserving endogenous PtdCho synthesis, which would blunt the compensatory drive for ETNPPL induction. ETNPPL likewise remained unchanged across choline levels under HF feeding in both sexes, in keeping with the obesogenic phenotype overriding choline-dependent regulation and with reports that prolonged HF feeding suppresses hepatic ETNPPL expression [65]. The induction of ETNPPL, and the insulin resistance that accompanied it, were therefore confined to choline-restricted males on the control diet, the same context in which the whole-animal phenotype emerged, positioning ETNPPL as a context-gated link between choline availability and hepatic insulin sensitivity.

In parallel, *Cd36* expression was increased at the mRNA level, suggesting enhanced dietary lipid sensing and uptake [66]. Although Cd36 protein abundance was unchanged, this pattern is consistent with nutrient-driven mRNA-protein discordance observed across metabolic pathways, where transcriptional responses occur without immediate changes in protein abundance and its role as a lipid sensor [67,68] regulated by substrate availability suggests that altered lipid flux contributes to the observed phenotype. The concurrent induction of Cd36, a well-established mediator whose chronic overexpression promotes pathological lipid uptake and insulin resistance [69], supports the interpretation that altered fatty acid flux contributes to the metabolic phenotype observed in ConLC mice. Together, these data support a model in which chronic low choline intake disrupts phospholipid balance, engages Etnppl-associated metabolic remodeling, and contributes to hepatic insulin resistance. These findings identify sustained low choline intake as a previously underappreciated driver of hepatic metabolic dysfunction, revealing a CD36–ETNPPL axis linking choline availability to phospholipid remodeling and insulin resistance. By demonstrating that insufficiency of a single essential nutrient can reprogram hepatic lipid architecture and endocrine signaling in the absence of diet-induced obesity, this work expands the framework through which choline intake is considered in metabolic disease and highlights phospholipid homeostasis as a regulator of hepatic insulin sensitivity.

CD36 has been implicated as a driver of obesity-associated metabolic dysfunction, where sustained upregulation promotes fatty acid uptake, ectopic lipid deposition, and insulin resistance [70,71]. In our model, Cd36 increased at the transcript level without a corresponding change in protein abundance, a pattern consistent with transcriptional priming under nutrient stress rather than requiring parallel changes in total protein abundance. This places CD36 as a responsive node engaged at the level of lipid sensing and flux adaptation, rather than a fully engaged driver in the low choline condition. Within this framework, CD36-mediated lipid handling represents a shared pathway engaged under both low choline and obese states. In obesity, however, chronic CD36 activation is associated with pathological lipid overload, consistent with the interpretation that this axis is already more strongly engaged and therefore less sensitive to modulation by choline availability. We did not directly test whether the CD36–ETNPPL axis is saturated in obese mice, and this should be considered a mechanistic possibility rather than a demonstrated outcome.

Beyond the mechanistic insights uncovered in this study, our findings highlight a broader but often overlooked dimension of metabolic disease: the role of choline, and other micronutrients, in shaping metabolic resilience. While obesity-driven metabolic dysfunction has traditionally been attributed to excess caloric intake, emerging evidence suggests that inadequate choline intake can disrupt metabolic homeostasis [[72], [73], [74]]. This concept is increasingly relevant in the context of modern therapeutic approaches such as GLP-1 receptor agonists, which profoundly alter micronutrient absorption [75]. Our findings suggest that sustained reductions in dietary choline may represent one such vulnerability, capable of causing insulin resistance even in the absence of diet-induced obesity, raising a critical translational question for the field: should assessment of choline status be incorporated into the metabolic evaluation of individuals at risk for insulin resistance and liver disease?

This study has several limitations arising from the emerging but still undefined role of ETNPPL in hepatic metabolism. Although our findings identify *Etnppl* as a transcriptional responder to sustained low choline intake and link both its expression and protein abundance to hepatic lipid remodeling, we did not directly measure lipid flux or phospholipid turnover under gain- or loss-of-function conditions. Thus, the specific metabolic pathways through which ETNPPL contributes to lipid remodeling remain to be defined. In addition, the causal contribution of ETNPPL to the development of insulin resistance remains to be directly tested in future studies using genetic or pharmacological approaches. While *Cd3*6 mRNA was increased under low choline conditions, CD36 protein abundance was unchanged, and we did not directly assess CD36 activity. Therefore, its role in lipid sensing in this context remains inferred from transcriptional activation and known regulation by substrate availability rather than direct functional measurement.

## 5 Conclusion

In summary, insufficiency of a single essential nutrient is enough to reprogram hepatic lipid metabolism and drive insulin resistance in male mice, independent of obesity. By tracing this phenotype to a choline-sensitive CD36–ETNPPL axis that links phospholipid balance to hepatic insulin sensitivity, our findings reframe dietary choline as an underappreciated and modifiable factor in metabolic disease.

## CRediT authorship contribution statement

**Evan M. Paules:** Writing – original draft, Visualization, Investigation, Formal analysis, Conceptualization. **Blake Rushing:** Formal analysis. **Israel Aguilar-Ordoñez:** Formal analysis. **Jose L. Garduno-Hernandez:** Methodology. **Ariana Reid:** Methodology. **Barbara J. Davis:** Formal analysis. **Teodoro Bottiglieri:** Formal analysis. **Karel Kalecký:** Formal analysis. **Erik Lopez-Gallardo:** Investigation. **Stephen D. Hursting:** Writing – review & editing, Supervision. **Isis Trujillo-Gonzalez:** Writing – review & editing, Writing – original draft, Supervision, Project administration, Conceptualization.

## Declaration of competing interest

EMP is a Balchem Postdoctoral Fellow. I.T-G has received research funding from Balchem. Balchem had no role in the study design; collection, analysis, or interpretation of data; writing of the manuscript; or decision to submit for publication.

## Figures and Tables

**Figure:**
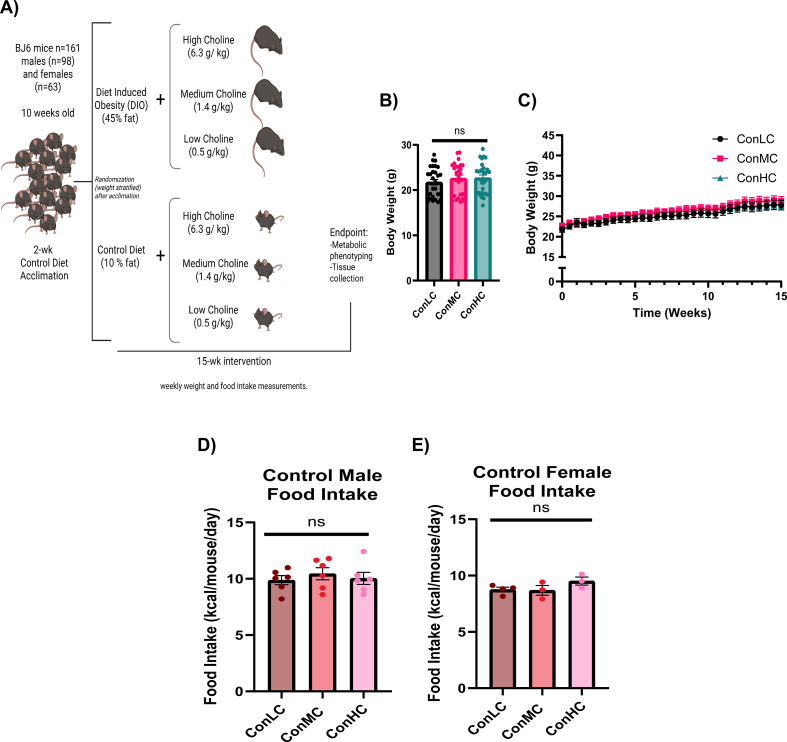
figs1

## Data Availability

Data will be made available on request.
