# Supplementary material for: Chronic choline restriction remodels hepatic lipid metabolism and drives insulin resistance through a CD36-ETNPPL regulatory axis
Source: Mol Metab. 2026 Jul 14;110:102411. doi: 10.1016/j.molmet.2026.102411 (PMC13382129; doi:10.1016/j.molmet.2026.102411)
Supplement: Multimedia component 1 [file mmc1.pdf]

RESEARCH DIETS INC.
www.ResearchDiets.com

Where NutriPhenomicsTM Begins

OpenSource DIETS™ BioDAQ®

CERTIFICATE OF CONFORMANCE

June 5, 2026

The diet product listed below has met our Quality Control standards at every stage of production, from formulation to packaging. The box has been sealed at the factory in New Brunswick, New Jersey, USA. Any repackaging or manipulation of the product invalidates this certificate.

Product #	Lot #	Diet Form	Package Type	Expiration Date
D22061504	22071303A1	½" Pellets	Standard	January 31, 2023

For further information, please feel free to contact us at 732-247-2390.

Thank you,

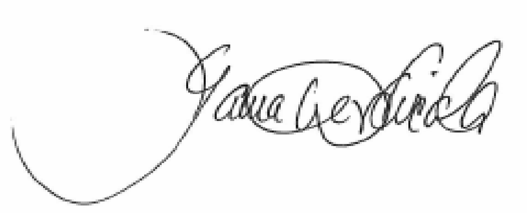

Laura DeNicola
Quality Assurance Officer | **Research Diets, Inc.**
20 Jules Lane | New Brunswick, NJ | 08901 USA
732.247.2390, Ext. 1481
email: LDeNicola@researchdiets.com
website: www.ResearchDiets.com
Learn about our BioDAQ food and water intake monitor.

RESEARCH DIETS INC.
www.ResearchDiets.com

Where NutriPhenomics™ Begins

OpenSource DIETS™ BioDAQ®

CERTIFICATE OF CONFORMANCE

June 5, 2026

The diet product listed below has met our Quality Control standards at every stage of production, from formulation to packaging. The box has been sealed at the factory in New Brunswick, New Jersey, USA. Any repackaging or manipulation of the product invalidates this certificate.

Product #	Lot #	Diet Form	Package Type	Expiration Date
D22061505	22071303A2	½" Pellets	Standard	January 31, 2023

For further information, please feel free to contact us at 732-247-2390.

Thank you,

Laura DeNicola
Quality Assurance Officer | **Research Diets, Inc.**
20 Jules Lane | New Brunswick, NJ | 08901 USA
732.247.2390, Ext. 1481
email: LDeNicola@researchdiets.com
website: www.ResearchDiets.com

Learn about our BioDAQ food and water intake monitor.

RESEARCH DIETS INC.
www.ResearchDiets.com

Where NutriPhenomics™ Begins

OpenSource DIETS™ BioDAQ®

CERTIFICATE OF CONFORMANCE

June 5, 2026

The diet product listed below has met our Quality Control standards at every stage of production, from formulation to packaging. The box has been sealed at the factory in New Brunswick, New Jersey, USA. Any repackaging or manipulation of the product invalidates this certificate.

Product #	Lot #	Diet Form	Package Type	Expiration Date
D22061506	22071303A3	½" Pellets	Standard	January 31, 2023

For further information, please feel free to contact us at 732-247-2390.

Thank you,

Laura DeNicola
Quality Assurance Officer | **Research Diets, Inc.**
20 Jules Lane | New Brunswick, NJ | 08901 USA
732.247.2390, Ext. 1481
email: LDeNicola@researchdiets.com
website: www.ResearchDiets.com

Learn about our BioDAQ food and water intake monitor.

CERTIFICATE OF CONFORMANCE

June 5, 2026

The diet product listed below has met our Quality Control standards at every stage of production, from formulation to packaging. The box has been sealed at the factory in New Brunswick, New Jersey, USA. Any repackaging or manipulation of the product invalidates this certificate.

**Product #**	**Lot #**	**Diet Form**	**Package Type**	**Expiration Date**
D22061507	22071303A4	½" Pellets	Standard	January 31, 2023

For further information, please feel free to contact us at 732-247-2390.

Thank you,

Laura DeNicola
Quality Assurance Officer | **Research Diets, Inc.**
20 Jules Lane | New Brunswick, NJ | 08901 USA
732.247.2390, Ext. 1481
email: LDeNicola@researchdiets.com
website: www.ResearchDiets.com
Learn about our BioDAQ food and water intake monitor.

Where NutriPhenomics™ Begins

CERTIFICATE OF CONFORMANCE

June 5, 2026

The diet product listed below has met our Quality Control standards at every stage of production, from formulation to packaging. The box has been sealed at the factory in New Brunswick, New Jersey, USA. Any repackaging or manipulation of the product invalidates this certificate.

Product #	Lot #	Diet Form	Package Type	Expiration Date
D22061508	22071303A5	½" Pellets	Standard	January 31, 2023

For further information, please feel free to contact us at 732-247-2390.

Thank you,

Laura DeNicola
Quality Assurance Officer | **Research Diets, Inc.**
20 Jules Lane | New Brunswick, NJ | 08901 USA
732.247.2390, Ext. 1481
email: LDeNicola@researchdiets.com
website: www.ResearchDiets.com

Learn about our BioDAQ food and water intake monitor.

CERTIFICATE OF CONFORMANCE

June 5, 2026

The diet product listed below has met our Quality Control standards at every stage of production, from formulation to packaging. The box has been sealed at the factory in New Brunswick, New Jersey, USA. Any repackaging or manipulation of the product invalidates this certificate.

Product #	Lot #	Diet Form	Package Type	Expiration Date
D22061509	22071303A6	½" Pellets	Standard	January 31, 2023

For further information, please feel free to contact us at 732-247-2390.

Thank you,

Laura DeNicola
Quality Assurance Officer | **Research Diets, Inc.**
20 Jules Lane | New Brunswick, NJ | 08901 USA
732.247.2390, Ext. 1481
email: LDeNicola@researchdiets.com
website: www.ResearchDiets.com
Learn about our BioDAQ food and water intake monitor.

CERTIFICATE OF CONFORMANCE

June 5, 2026

The diet product listed below has met our Quality Control standards at every stage of production, from formulation to packaging. The box has been sealed at the factory in New Brunswick, New Jersey, USA. Any repackaging or manipulation of the product invalidates this certificate.

Product #	Lot #	Diet Form	Package Type	Expiration Date
D22061504	23081009A1	½" Pellets	Standard	February 29, 2024

For further information, please feel free to contact us at 732-247-2390.

Thank you,

Laura DeNicola
Quality Assurance Officer | **Research Diets, Inc.**
20 Jules Lane | New Brunswick, NJ | 08901 USA
732.247.2390, Ext. 1481
email: LDeNicola@researchdiets.com
website: www.ResearchDiets.com
Learn about our BioDAQ food and water intake monitor.

CERTIFICATE OF CONFORMANCE

June 5, 2026

The diet product listed below has met our Quality Control standards at every stage of production, from formulation to packaging. The box has been sealed at the factory in New Brunswick, New Jersey, USA. Any repackaging or manipulation of the product invalidates this certificate.

Product #	Lot #	Diet Form	Package Type	Expiration Date
D22061505	23081009A2	½" Pellets	Standard	February 29, 2024

For further information, please feel free to contact us at 732-247-2390.

Thank you,

Laura DeNicola
Quality Assurance Officer | **Research Diets, Inc.**
20 Jules Lane | New Brunswick, NJ | 08901 USA
732.247.2390, Ext. 1481
email: LDeNicola@researchdiets.com
website: www.ResearchDiets.com
Learn about our BioDAQ food and water intake monitor.

RESEARCH DIETS INC.
www.ResearchDiets.com

Where NutriPhenomics™ Begins

OpenSource DIETS™ BioDAQ®

CERTIFICATE OF CONFORMANCE

June 5, 2026

The diet product listed below has met our Quality Control standards at every stage of production, from formulation to packaging. The box has been sealed at the factory in New Brunswick, New Jersey, USA. Any repackaging or manipulation of the product invalidates this certificate.

Product #	Lot #	Diet Form	Package Type	Expiration Date
D22061506	23081009A3	½" Pellets	Standard	February 29, 2024

For further information, please feel free to contact us at 732-247-2390.

Thank you,

Laura DeNicola
Quality Assurance Officer | **Research Diets, Inc.**
20 Jules Lane | New Brunswick, NJ | 08901 USA
732.247.2390, Ext. 1481
email: LDeNicola@researchdiets.com
website: www.ResearchDiets.com
Learn about our BioDAQ food and water intake monitor.

Research Diets, Inc. • 20 Jules Lane • New Brunswick, NJ USA
P: 732-247-2390 • F: 732-247-2340 • info@researchdiets.com

CERTIFICATE OF CONFORMANCE

June 5, 2026

The diet product listed below has met our Quality Control standards at every stage of production, from formulation to packaging. The box has been sealed at the factory in New Brunswick, New Jersey, USA. Any repackaging or manipulation of the product invalidates this certificate.

Product #	Lot #	Diet Form	Package Type	Expiration Date
D22061507	23081009A4	½” Pellets	Standard	February 29, 2024

For further information, please feel free to contact us at 732-247-2390.

Thank you,

Laura DeNicola
Quality Assurance Officer | **Research Diets, Inc.**
20 Jules Lane | New Brunswick, NJ | 08901 USA
732.247.2390, Ext. 1481
email: LDeNicola@researchdiets.com
website: www.ResearchDiets.com
Learn about our BioDAQ food and water intake monitor.

CERTIFICATE OF CONFORMANCE

June 5, 2026

The diet product listed below has met our Quality Control standards at every stage of production, from formulation to packaging. The box has been sealed at the factory in New Brunswick, New Jersey, USA. Any repackaging or manipulation of the product invalidates this certificate.

Product #	Lot #	Diet Form	Package Type	Expiration Date
D22061508	23081009A5	½" Pellets	Standard	February 29, 2024

For further information, please feel free to contact us at 732-247-2390.

Thank you,

Laura DeNicola
Quality Assurance Officer | **Research Diets, Inc.**
20 Jules Lane | New Brunswick, NJ | 08901 USA
732.247.2390, Ext. 1481
email: LDeNicola@researchdiets.com
website: www.ResearchDiets.com
Learn about our BioDAQ food and water intake monitor.

CERTIFICATE OF CONFORMANCE

June 5, 2026

The diet product listed below has met our Quality Control standards at every stage of production, from formulation to packaging. The box has been sealed at the factory in New Brunswick, New Jersey, USA. Any repackaging or manipulation of the product invalidates this certificate.

Product #	Lot #	Diet Form	Package Type	Expiration Date
D22061509	23081009A6	½" Pellets	Standard	February 29, 2024

For further information, please feel free to contact us at 732-247-2390.

Thank you,

Laura DeNicola
Quality Assurance Officer | **Research Diets, Inc.**
20 Jules Lane | New Brunswick, NJ | 08901 USA
732.247.2390, Ext. 1481
email: LDeNicola@researchdiets.com
website: www.ResearchDiets.com
Learn about our BioDAQ food and water intake monitor.

CERTIFICATE OF CONFORMANCE

June 5, 2026

The diet product listed below has met our Quality Control standards at every stage of production, from formulation to packaging. The box has been sealed at the factory in New Brunswick, New Jersey, USA. Any repackaging or manipulation of the product invalidates this certificate.

Product #	Lot #	Diet Form	Package Type	Expiration Date
D22061505	23102803A1	½" Pellets	Standard	April 30, 2024

For further information, please feel free to contact us at 732-247-2390.

Thank you,

Laura DeNicola
Quality Assurance Officer | **Research Diets, Inc.**
20 Jules Lane | New Brunswick, NJ | 08901 USA
732.247.2390, Ext. 1481
email: LDeNicola@researchdiets.com
website: www.ResearchDiets.com
Learn about our BioDAQ food and water intake monitor.

CERTIFICATE OF CONFORMANCE

June 5, 2026

The diet product listed below has met our Quality Control standards at every stage of production, from formulation to packaging. The box has been sealed at the factory in New Brunswick, New Jersey, USA. Any repackaging or manipulation of the product invalidates this certificate.

Product #	Lot #	Diet Form	Package Type	Expiration Date
D22061508	23102803A2	½" Pellets	Standard	April 30, 2024

For further information, please feel free to contact us at 732-247-2390.

Thank you,

Laura DeNicola
Quality Assurance Officer | **Research Diets, Inc.**
20 Jules Lane | New Brunswick, NJ | 08901 USA
732.247.2390, Ext. 1481
email: LDeNicola@researchdiets.com
website: www.ResearchDiets.com
Learn about our BioDAQ food and water intake monitor.

CERTIFICATE OF CONFORMANCE

June 8, 2026

The diet product listed below has met our Quality Control standards at every stage of production, from formulation to packaging. The box has been sealed at the factory in New Brunswick, New Jersey, USA. Any repackaging or manipulation of the product invalidates this certificate.

Product #	Lot #	Diet Form	Package Type	Expiration Date
D22061505	23121913	½" Pellets	Standard	June 30, 2024

For further information, please feel free to contact us at 732-247-2390.

Thank you,

Laura DeNicola
Quality Assurance Officer | **Research Diets, Inc.**
20 Jules Lane | New Brunswick, NJ | 08901 USA
732.247.2390, Ext. 1481
email: LDeNicola@researchdiets.com
website: www.ResearchDiets.com
Learn about our BioDAQ food and water intake monitor.

CERTIFICATE OF CONFORMANCE

June 5, 2026

The diet product listed below has met our Quality Control standards at every stage of production, from formulation to packaging. The box has been sealed at the factory in New Brunswick, New Jersey, USA. Any repackaging or manipulation of the product invalidates this certificate.

Product #	Lot #	Diet Form	Package Type	Expiration Date
D22061508	23012601	½" Pellets	Standard	July 31, 2023

For further information, please feel free to contact us at 732-247-2390.

Thank you,

Laura DeNicola
Quality Assurance Officer | **Research Diets, Inc.**
20 Jules Lane | New Brunswick, NJ | 08901 USA
732.247.2390, Ext. 1481
email: LDeNicola@researchdiets.com
website: www.ResearchDiets.com
Learn about our BioDAQ food and water intake monitor.

RESEARCH DIETS INC.
www.ResearchDiets.com

Where NutriPhenomics™ Begins

OpenSource DIETS™ BioDAQ®

CERTIFICATE OF CONFORMANCE

June 8, 2026

The diet product listed below has met our Quality Control standards at every stage of production, from formulation to packaging. The box has been sealed at the factory in New Brunswick, New Jersey, USA. Any repackaging or manipulation of the product invalidates this certificate.

Product #	Lot #	Diet Form	Package Type	Expiration Date
D22061504	24021509A1	½” Pellets	Standard	August 31, 2024

For further information, please feel free to contact us at 732-247-2390.

Thank you,

Laura DeNicola
Quality Assurance Officer | **Research Diets, Inc.**
20 Jules Lane | New Brunswick, NJ | 08901 USA
732.247.2390, Ext. 1481
email: LDeNicola@researchdiets.com
website: www.ResearchDiets.com
Learn about our BioDAQ food and water intake monitor.

RESEARCH DIETS INC.
www.ResearchDiets.com

Where NutriPhenomics™ Begins

OpenSource DIETS™ BioDAQ®

CERTIFICATE OF CONFORMANCE

June 8, 2026

The diet product listed below has met our Quality Control standards at every stage of production, from formulation to packaging. The box has been sealed at the factory in New Brunswick, New Jersey, USA. Any repackaging or manipulation of the product invalidates this certificate.

Product #	Lot #	Diet Form	Package Type	Expiration Date
D22061506	24021509A2	½" Pellets	Standard	August 31, 2024

For further information, please feel free to contact us at 732-247-2390.

Thank you,

Laura DeNicola
Quality Assurance Officer | **Research Diets, Inc.**
20 Jules Lane | New Brunswick, NJ | 08901 USA
732.247.2390, Ext. 1481
email: LDeNicola@researchdiets.com
website: www.ResearchDiets.com
Learn about our BioDAQ food and water intake monitor.

CERTIFICATE OF CONFORMANCE

June 8, 2026

The diet product listed below has met our Quality Control standards at every stage of production, from formulation to packaging. The box has been sealed at the factory in New Brunswick, New Jersey, USA. Any repackaging or manipulation of the product invalidates this certificate.

Product #	Lot #	Diet Form	Package Type	Expiration Date
D22061507	24021509A3	½" Pellets	Standard	August 31, 2024

For further information, please feel free to contact us at 732-247-2390.

Thank you,

Laura DeNicola
Quality Assurance Officer | **Research Diets, Inc.**
20 Jules Lane | New Brunswick, NJ | 08901 USA
732.247.2390, Ext. 1481
email: LDeNicola@researchdiets.com
website: www.ResearchDiets.com
Learn about our BioDAQ food and water intake monitor.

OpenSource DIETS™ BioDAQ®

CERTIFICATE OF CONFORMANCE

June 8, 2026

The diet product listed below has met our Quality Control standards at every stage of production, from formulation to packaging. The box has been sealed at the factory in New Brunswick, New Jersey, USA. Any repackaging or manipulation of the product invalidates this certificate.

Product #	Lot #	Diet Form	Package Type	Expiration Date
D22061509	24021509A4	½" Pellets	Standard	August 31, 2024

For further information, please feel free to contact us at 732-247-2390.

Thank you,

Laura DeNicola
Quality Assurance Officer | **Research Diets, Inc.**
20 Jules Lane | New Brunswick, NJ | 08901 USA
732.247.2390, Ext. 1481
email: LDeNicola@researchdiets.com
website: www.ResearchDiets.com
Learn about our BioDAQ food and water intake monitor.

CERTIFICATE OF CONFORMANCE

June 8, 2026

The diet product listed below has met our Quality Control standards at every stage of production, from formulation to packaging. The box has been sealed at the factory in New Brunswick, New Jersey, USA. Any repackaging or manipulation of the product invalidates this certificate.

Product #	Lot #	Diet Form	Package Type	Expiration Date
D22061505	24050615A1	½" Pellets	Standard	November 30, 2024

For further information, please feel free to contact us at 732-247-2390.

Thank you,

Laura DeNicola
Quality Assurance Officer | **Research Diets, Inc.**
20 Jules Lane | New Brunswick, NJ | 08901 USA
732.247.2390, Ext. 1481
email: LDeNicola@researchdiets.com
website: www.ResearchDiets.com
Learn about our BioDAQ food and water intake monitor.

OpenSource DIETS™ BioDAQ®

CERTIFICATE OF CONFORMANCE

June 8, 2026

The diet product listed below has met our Quality Control standards at every stage of production, from formulation to packaging. The box has been sealed at the factory in New Brunswick, New Jersey, USA. Any repackaging or manipulation of the product invalidates this certificate.

Product #	Lot #	Diet Form	Package Type	Expiration Date
D22061508	24050615A2	½" Pellets	Standard	November 30, 2024

For further information, please feel free to contact us at 732-247-2390.

Thank you,

Laura DeNicola
Quality Assurance Officer | **Research Diets, Inc.**
20 Jules Lane | New Brunswick, NJ | 08901 USA
732.247.2390, Ext. 1481
email: LDeNicola@researchdiets.com
website: www.ResearchDiets.com
Learn about our BioDAQ food and water intake monitor.
