# Supplementary material for: Chronic choline restriction remodels hepatic lipid metabolism and drives insulin resistance through a CD36-ETNPPL regulatory axis
Source: Mol Metab. 2026 Jul 14;110:102411. doi: 10.1016/j.molmet.2026.102411 (PMC13382129; doi:10.1016/j.molmet.2026.102411)
Supplement: Multimedia component 2 [file mmc2.docx]

|  | **HFLC** | | **HFMC** | | **HFHC** | | **ConLC** | | **ConMC** | | **ConHC** | |
| --- | --- | --- | --- | --- | --- | --- | --- | --- | --- | --- | --- | --- |
|  | **gm (%)** | **Kcal (%)** | **gm (%)** | **Kcal (%)** | **gm (%)** | **Kcal (%)** | **gm (%)** | **Kcal (%)** | **gm (%)** | **Kcal (%)** | **gm (%)** | **Kcal (%)** |
| Protein | 24 | 20 | 24 | 20 | 24 | 20 | 19 | 20 | 19 | 20 | 19 | 20 |
| Carbohydrate | 41 | 35 | 41 | 35 | 41 | 35 | 67 | 70 | 67 | 70 | 67 | 70 |
| Fat | 24 | 45 | 24 | 45 | 24 | 45 | 4 | 10 | 4 | 10 | 4 | 10 |
| Total | 4.7 | 100 | 4.7 | 100 | 4.7 | 100 | 3.8 | 100 | 3.8 | 100 | 3.8 | 100 |
|  |  |  |  |  |  |  |  |  |  |  |  |  |
| **Ingredient** | **gm/Kg** | **Kcal/Kg** | **gm/Kg** | **Kcal/Kg** | **gm/Kg** | **Kcal/Kg** | **gm/Kg** | **Kcal/Kg** | **gm/Kg** | **Kcal/Kg** | **gm/Kg** | **Kcal/Kg** |
| Casein | 200 | 800 | 200 | 800 | 200 | 800 | 200 | 800 | 200 | 800 | 200 | 800 |
| L-Cystine | 3 | 12 | 3 | 12 | 3 | 12 | 3 | 12 | 3 | 12 | 3 | 12 |
| Cornstarch | 72.8 | 291 | 72.8 | 291 | 72.8 | 291 | 452.2 | 1809 | 452.2 | 1809 | 452.2 | 1809 |
| Maltodextrin 10 | 100 | 400 | 100 | 400 | 100 | 400 | 75 | 300 | 75 | 300 | 75 | 300 |
| Sucrose | 172.8 | 691 | 172.8 | 691 | 172.8 | 691 | 172.8 | 691 | 172.8 | 691 | 172.8 | 691 |
| Cellulose, BW200 | 50 | 0 | 50 | 0 | 50 | 0 | 50 | 0 | 50 | 0 | 50 | 0 |
| Soybean Oil | 25 | 225 | 25 | 225 | 25 | 225 | 25 | 225 | 25 | 225 | 25 | 225 |
| Lard | 177.5 | 1598 | 177.5 | 1598 | 177.5 | 1598 | 20 | 180 | 20 | 180 | 20 | 180 |
| Mineral Mix | 10 | 0 | 10 | 0 | 10 | 0 | 10 | 0 | 10 | 0 | 10 | 0 |
| Vitamin Mix | 10 | 0 | 10 | 0 | 10 | 0 | 10 | 0 | 10 | 0 | 10 | 0 |
| Choline Chloride | 0.5 | 0 | 1.4 | 0 | 6.3 | 0 | 0.5 | 0 | 1.4 | 0 | 6.3 | 0 |

Supplementary Table 1

Gram (gm)
