# Supplementary material for: Chronic choline restriction remodels hepatic lipid metabolism and drives insulin resistance through a CD36-ETNPPL regulatory axis
Source: Mol Metab. 2026 Jul 14;110:102411. doi: 10.1016/j.molmet.2026.102411 (PMC13382129; doi:10.1016/j.molmet.2026.102411)
Supplement: Multimedia component 3 [file mmc3.docx]

Supplementary Table 2

| **Figure** | **Outcome** | **Test** | **Comparison/ Effect** | **Omnibus Statistic** | **Post hoc Statistic** | **Adjusted p** |
| --- | --- | --- | --- | --- | --- | --- |
| 1A | Body weight over time | Mixed-Effects Model | Time x diet interaction | F (60, 1356) = 2.28, p < 0.0001 | - | - |
| 1B | Body weight change | Kruskal-Wallis One-Way ANOVA, Dunn’s | ConLC vs ConHC | H (2) = 8.005, p = 0.0183 | z = 2.753 | p = 0.0177 |
| 1C | Body weight over time | Mixed-Effects Model | Time | F (30, 885) = 87.26, p < 0.0001 | - | - |
| 1E | Fat mass | Two-Way ANOVA, Tukey’s | ConLC vs ConHC | F (2, 46) = 4.606, p = 0.0150 | q (92) = 4.249 | p = 0.0095 |
| 1F | Lean/Fat mass ratio | Kruskal-Wallis One-Way ANOVA, Dunn’s | ConLC vs ConHC | H (2) = 12.7, p = 0.0017 | z = 3.55 | p = 0.0011 |
| 1G | Lean mass | Two-Way ANOVA, Tukey’s | ConLC vs ConHC | F (2, 30) = 8.563, p = 0.0011 | q (60) = 4.738 | p = 0.0040 |
| 1G | Lean mass | Two-Way ANOVA, Tukey’s | ConLC vs ConMC | F (2, 30) = 8.563, p = 0.0011 | q (60) = 4.071 | p = 0.0151 |
| 1H | Lean/Fat mass ratio | One-Way ANOVA, Tukey’s | ConLC vs ConMC | F (2, 30) = 3.180, p = 0.0559 | q (30) = 3.489 | p = 0.0498 |
| 2A | Leptin | Two-Way ANOVA, Tukey’s | ConLC vs ConMC | F (16, 135) = 5.090, p < 0.0001 | q (135) = 7.177 | p < 0.0001 |
| 2A | Leptin | Two-Way ANOVA, Tukey’s | ConLC vs ConHC | F (16, 135) = 5.090, p < 0.0001 | q (135) = 8.040 | p < 0.0001 |
| 2A | Ghrelin | Two-Way ANOVA, Tukey’s | ConLC vs ConMC | F (16, 135) = 5.090, p < 0.0001 | q (135) = 4.056 | p = 0.0132 |
| 2A | Ghrelin | Two-Way ANOVA, Tukey’s | ConLC vs ConHC | F (16, 135) = 5.090, p < 0.0001 | q (135) = 6.819 | p < 0.0001 |
| 2A | Adiponectin | Two-Way ANOVA, Tukey’s | ConLC vs ConMC | F (16, 135) = 5.090, p < 0.0001 | q (135) = 4.526 | p = 0.0048 |
| 2A | Adiponectin | Two-Way ANOVA, Tukey’s | ConLC vs ConHC | F (16, 135) = 5.090, p < 0.0001 | q (135) = 6.741 | p <0.001 |
| 2B | Log(adiponectin/Leptin) | One-Way ANOVA, Tukey’s | ConLC vs ConMC | F (2, 15) = 6.727, p = 0.0082 | q (15) = 3.993 | p = 0.0324 |
| 2B | Log(adiponectin/Leptin) | One-Way ANOVA, Tukey’s | ConLC vs ConHC | F (2, 15) = 6.727, p = 0.0082 | q (15) = 4.864 | p = 0.0096 |
| 2C | Ghrelin | Two-Way ANOVA, Tukey’s | ConLC vs ConMC | F (16, 135) = 1.916, P = 0.0238 | q (135) = 4.650 | p = 0.0037 |
| 2C | Adiponectin | Two-Way ANOVA, Tukey’s | ConLC vs ConMC | F (16, 135) = 1.916, P = 0.0238 | q (135) = 5.715 | p = 0.0003 |
| 2C | Adiponectin | Two-Way ANOVA, Tukey’s | ConLC vs ConHC | F (16, 135) = 1.916, P = 0.0238 | q (135) = 3.649 | p = 0.0293 |
| 3A | Body weight over time | Mixed-Effects Model | Time x diet interaction | F (60, 1358) = 2.137, p <0.0001 | - | - |
| 3A | Body weight over time | Mixed-Effects Model, Tukey’s | HFMC vs HFHC | F (60, 1358) = 2.137, p <0.0001 | q (1404) = 4.097 | p < 0.05 |
| 3B | Body weight change | One-way ANOVA, Tukey's | HFMC vs HFHC | F (2, 46) = 4.919, p = 0.0116 | q (46) = 4.395 | p = 0.0089 |
| 3C | Body weight over time | Mixed-Effects Model | Time | F (30, 804) = 103.1, p < 0.0001 | - | - |
| 3E | Fat mass | Two-Way ANOVA, Tukey’s | HFLC vs HFMC | F (2, 46) = 1.267, p = 0.2914 | q (92) = 3.546 | p = 0.0367 |
| 3E | Fat mass | Two-Way ANOVA, Tukey’s | HFMC vs HFHC | F (2, 46) = 1.267, P = 0.2914 | q (92) = 4.072 | p = 0.0136 |
| 4C | NAS | Two-way ANOVA, Tukey's | ConLC vs ConMC | F (2, 39) = 17.51, p < 0.001 | q (39) = 7.486 | p <0.0001 |
| 4C | NAS | Two-way ANOVA, Tukey's | ConLC vs ConHC | F (2, 39) = 17.51, p < 0.001 | q (39) = 6.695 | p <0.0001 |
| 5E | Hepatic TAG | Welch’s ANOVA,  Dunnett’s T3 | ConMC vs ConHC | W (2, 17.88) = 3.748, p = 0.0437 | t (19.95) = 2.657 | p = 0.0438 |
| 7D | Protein (ETNPPL) | One-way ANOVA, Fisher's LSD | ConLC vs ConMC | F (2, 33) = 4.229, p = 0.0232 | t (33) = 2.345 | p = 0.0252 |
| 7D | Protein (ETNPPL) | One-way ANOVA, Fisher's LSD | ConLC vs ConHC | F (2, 33) = 4.229, p = 0.0232 | t (33) = 2.662 | p = 0.0119 |
| 7E | Glucose over time | Two-way RM ANOVA, Fisher's LSD | ConLC vs ConHC | F (6, 138) = 0.925, p = 0.48 | t (184) = 2.526 | p <0.05 |
| 7E | Glucose over time | Two-way RM ANOVA, Fisher's LSD | ConLC vs ConMC | F (6, 138) = 0.925, p = 0.48 | t (184) = 2.168 | p <0.05 |
| 7E | Glucose over time | Two-way RM ANOVA, Fisher's LSD | ConLC vs ConHC | F (6, 138) = 0.925, p = 0.48 | t (184) = 2.330 | p <0.05 |
| 7F | HOMA-IR | One-way ANOVA, Fisher's LSD | ConLC vs ConMC | F (2, 13) = 10.77, p = 0.0017 | t (13) = 4.540 | p = 0.0006 |
| 7F | HOMA-IR | One-way ANOVA, Fisher's LSD | ConLC vs ConHC | F (2, 13) = 10.77, p = 0.0017 | t (13) = 3.195 | p = 0.0070 |
| SF 2A | gWAT weight | One-way ANOVA, Tukey's | ConLC vs ConMC | F (2, 46) = 6.395, p = 0.0035 | q (46) = 3.617 | p = 0.0364 |
| SF 2A | gWAT weight | One-way ANOVA, Tukey's | ConLC vs ConHC | F (2, 46) = 6.395, p = 0.0035 | q (46) = 4.917 | p = 0.0032 |
| SF 2F | Kidney weight | One-way ANOVA, Tukey's | ConLC vs ConHC | F (2, 30) = 6.084, p = 0.006 | q (30) = 4.529 | p = 0.009 |
| SF 2F | Kidney weight | One-way ANOVA, Tukey's | ConMC vs ConHC | F (2, 30) = 6.084, p = 0.006 | q (30) = 3.936 | p = 0.02 |
| SF 4B | Liver weight | Welch's ANOVA, Dunnett's T3 | HFMC vs HFHC | W (2, 27.43) = 12.39, p < 0.001 | t (22.92) = 4.873 | p = 0.0002 |
| SF 4C | Kidney weight | One-way ANOVA, Tukey's | HFMC vs HFHC | F (2, 46) = 4.515, p = 0.0162 | q (46) = 4.168 | p = 0.0137 |
| SF 4K | Leptin | Two-way ANOVA, Tukey's | HFLC vs HFMC | F (16, 120) = 1.263, p = 0.2319 | q (135) = 5.317 | p = 0.0007 |
| SF 4K | Leptin | Two-way ANOVA, Tukey's | HFLC vs HFHC | F (16, 120) = 1.263, p = 0.2319 | q (135) = 3.884 | p = 0.0187 |
| SF 4M | Insulin | Two-way ANOVA, Tukey's | HFMC vs HFHC | F (16, 119) = 1.114, p = 0.3499 | q (134) = 3.627 | p = 0.0305 |
| SF 4M | Adiponectin | Two-way ANOVA, Tukey's | HFMC vs HFHC | F (16, 119) = 1.114, p = 0.3499 | q (134) = 4.116 | p = 0.0117 |
| SF 5C | NAS | Two-way ANOVA, Tukey's | HFLC vs HFMC | F (2, 45) = 3.024, p = 0.0586 | q (45) = 3.478 | p = 0.0460 |
